# Meta-analysis of the effect of probiotics or synbiotics on the risk factors in patients with coronary artery disease

**DOI:** 10.3389/fcvm.2023.1154888

**Published:** 2023-08-02

**Authors:** Yunzhen Lei, Min Xu, Nanqu Huang, Zhengqiang Yuan

**Affiliations:** ^1^ Department of Cardiovascular Medicine, The Third Affiliated Hospital of Zunyi Medical University (The First People’s Hospital of Zunyi), Zunyi, China; ^2^ Drug Clinical Trial Institution, The Third Affiliated Hospital of Zunyi Medical University (The First People’s Hospital of Zunyi), Zunyi, China

**Keywords:** coronary artery disease, probiotics, synbiotics, meta-analysis, coronary atherosclerosis

## Abstract

**Objective:**

The objective of this study was to study the effect of probiotics or synbiotics on the risk factors for coronary artery disease (CAD) in the context of conventional drug therapy for CAD.

**Methods:**

The literature on probiotics or synbiotics for the treatment of CAD was collected from PubMed, Scopus, Web of Science, Embase, and Cochrane Library. The search period was conducted on November 5, 2022, and the search covered all literature before November 5, 2022. The included literature consisted of randomized controlled trials of probiotics or synbiotics for CAD, and a meta-analysis was performed using Stata 14 software and RevMan 5.4 software.

**Results:**

The meta-analysis explored the effect of probiotics or synbiotics on the risk factors for coronary artery lesions in a treatment setting with conventional medications for CAD. After a rigorous literature screening process, 10 studies were finally included for data consolidation to objectively evaluate the effect of probiotics or synbiotics on coronary lesions. The results of this study showed that the addition of probiotics or synbiotics to conventional medications for CAD reduced the levels of low-density lipoprotein cholesterol [weighted mean difference (WMD) −9.13 (−13.17, −5.09)], fasting glucose (FPG) [WMD −13.60 (−23.57, −3.62)], and hypersensitive C-reactive protein (hs-CRP) [standardized mean difference (SMD) −0.60 (−0.83, −0.37)] and increased the levels of high-density lipoprotein cholesterol (HDL-C) [WMD 1.94 (0.32, 3.57)], nitric oxide (NO) [WMD 5.38 (3.23, 7.54)] but did not affect the triglyceride (TG) level [WMD −13.41 (−28.03, 1.21)], systolic blood pressure (SBP) [WMD −0.88 (−3.72, 1.96)], or diastolic blood pressure (DBP) [WMD −0.21 (−2.19, 1.76)].

**Conclusion:**

Adding probiotics or synbiotics to conventional medications for CAD may improve patient prognosis.

**Systematic Review Registration:**

https://www.crd.york.ac.uk/PROSPERO/, identifier CRD42022362711.

## 1. Introduction

Coronary artery disease (CAD) is a cardiovascular disease characterized by a chronic inflammatory response and plaque accumulation, eventually leading to myocardial ischemia, hypoxia, or necrosis. It is one of the leading causes of death in patients. Hypertension, diabetes mellitus, hyperlipidemia, and long-term chronic inflammatory response are the important risk factors for CAD (1). Although conventional drugs for CAD have achieved good results in delaying coronary artery lesions, the morbidity and mortality of patients with CAD are still increasing (2). Therefore, further controlling the risk factors of coronary artery lesions is of great importance in improving the prognosis of patients.

Probiotics or synbiotics are living microorganisms and are used as non-invasive therapeutic tools. They have been found to delay the progression of coronary artery lesions through the “entero-cardiac axis,” providing new insights to improve the prognosis of patients with CAD (3, 4). The use of probiotics or synbiotics to modulate the gut microbiota in patients with CAD may reduce the risk factors for CAD, such as hypersensitive C-reactive protein (hs-CRP), nitric oxide (NO), low-density lipoprotein cholesterol (LDL-C), and total cholesterol (TC) (5, 6). However, some studies have yielded different results (7, 8), and there is a lack of evidence-based medical proof. Therefore, the present meta-analysis included a population of patients with CAD and integrated all relevant studies on using probiotics or synbiotics to reduce the risk factors for coronary artery lesions, beginning in November 2022. The aim is to provide a theoretical basis for probiotics or synbiotics to further improve the prognosis of patients with CAD.

## 2. Materials and methods

### 2.1. Protocols

This systematic review and meta-analysis was conducted strictly in accordance with the protocol registered in the PROSPERO (CRD42022362711) and PRISMA guidelines.

### 2.2. Search criteria

#### 2.2.1. Inclusion criteria

The inclusion criteria include the design using the PICOS principles: (1) study subjects (P): patients with CAD using probiotics or synbiotics; (2) intervention (I): probiotics or synbiotics; (3) control measures (C): patients with CAD not using probiotics or synbiotics; (4) outcome indicators (O): TC, triglycerides (TGs), LDL-C, high-density lipoprotein cholesterol (HDL-C), very low-density lipoprotein (VLDL), TC-to-high-density lipoprotein cholesterol ratio (TOTAL-/HDL-C%), fasting glucose (FPG), insulin, insulin sensitivity index (QUICKI), insulin resistance index (HOMA-IR), systolic blood pressure (SBP), diastolic blood pressure (DBP), trimethylamine oxide (TMAO), lipopolysaccharide (LPS), ultrasensitive C-reactive protein (hs-CRP), glutathione (GSH), NO, and total antioxidant capacity (TAC); and (5) study type (S): randomized controlled trials (RCTs).

#### 2.2.2. Exclusion criteria

The exclusion criteria are as follows: (1) animal experiments; (2) reviews and case reports; (3) inaccessible valid data; (4) duplicate published papers; and (5) patients with severe hepatic and renal insufficiency.

### 2.3. Search databases

PubMed, Scopus, Web of Science, Embase, and Cochrane library were searched from their respective establishment dates on November 5, 2022, and the scope of the search was all the literature before November 5, 2022. The search strategy is shown in Supplementary Table S1.

### 2.4. Search strategy, data extraction, and quality assessment

Two researchers independently screened the literature and extracted the data based on the established inclusion and exclusion criteria. The literature was initially screened by reading the titles and abstracts, and those not meeting the inclusion criteria were excluded. The remaining articles were then read to determine their final inclusion. Any disagreements were resolved through discussion among all researchers. Regarding the quality assessment of the literature, the final included RCTs were evaluated by two investigators using a bias assessment tool. This tool assessed randomization, allocation concealment, blinding, incomplete results, selective reporting, and other potential risks. Any disagreements were resolved through discussion among all investigators. Finally, the evidence contained within the article was rated using “grade grading” (Supplementary Table S3).

### 2.5. Statistical analysis

The meta-analysis was performed using Stata 14.0 software and RevMan 5.4 software. The sample size, mean, and variance before and after the intervention were extracted. The means and standard deviations of changes in outcome variables before and after the intervention were calculated according to the method provided in the Cochrane Handbook 5.0.2 (16.1.3.2) using the following formula: $\mathrm{Mean}{\;}_{E,\;\mathrm{change}}=\mathrm{Mean}{\;}_{E,\;\mathrm{final}}-\mathrm{Mean}{\;}_{E,\;\mathrm{baseline}}$ $SD_{E,\mathrm{change}}=$ $\;\sqrt{{\mathrm{SD}}_{E,b\mathrm{aseline}}^{2}+{\mathrm{SD}}_{E,\mathrm{final}}^{2}-(2*\mathrm{Corr}*SD_{E,\;\mathrm{baseline}}*SD_{E,\mathrm{final}})}$, Corr = 0.50

The statistical heterogeneity of the included studies was analyzed using the *Q* test and *I*^2^ test. *I*^2 ^< 50% was considered low heterogeneity, and *I*^2 ^> 50% was considered high heterogeneity. A fixed-effects model was used if there was no statistical heterogeneity among the findings. In case of significant clinical heterogeneity, methods such as subgroup analysis or sensitivity analysis were used to address it (Supplementary Figure S1). A funnel plot was used to evaluate the publication bias of the literature (Supplementary Figure S2).

## 3. Results

### 3.1. Literature search results

**Figure 1:**
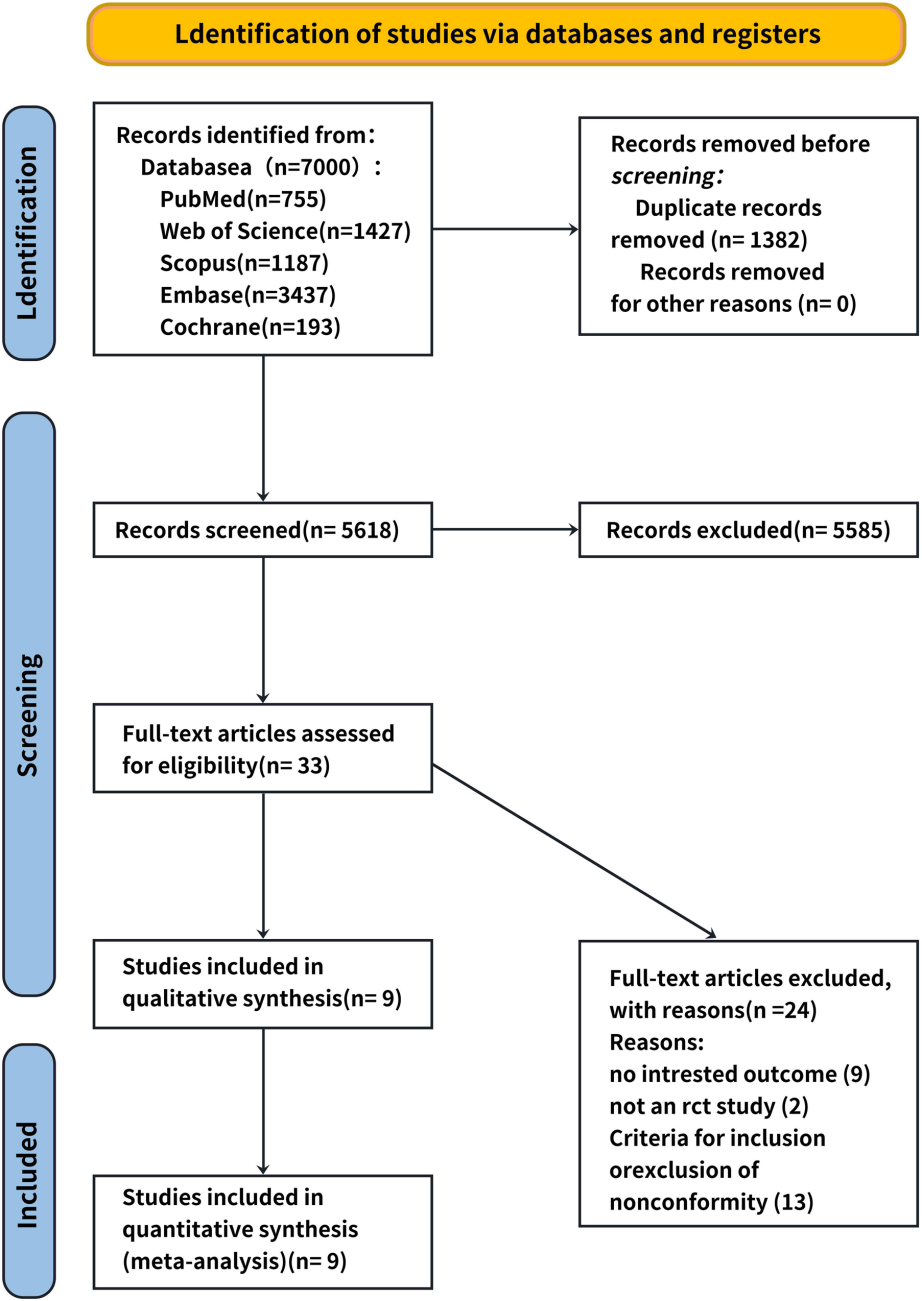
Literature screening processes and results.

A total of 34 relevant studies were obtained in the preliminary examination, and 10 records were finally included after screening. Nine records were eliminated because they did not have specific values, two records were excluded because they were not RCT experiments, and 13 records were excluded because they did not comply with the inclusion and exclusion criteria of this systematic review. The literature screening processes and results are shown in Figure 1.

### 3.2. Description of included trials

All articles were RCTs and included values for TC, TG, LDL-C, HDL-C, VLDL, TOTAL-/HDL-C%, FPG, insulin, QUICKI, HOMA-IR, SBP, DBP, TMAO, hs-CRP, GSH, NO, TAC, and beneficial bacteria species including *Lactobacillus acidophilus*, *Bifidobacterium bifidum*, *Lactobacillus reuteri*, *Lactobacillus fermentum*, and *Lactobacillus rhamnosus*. The details of the study characteristics are presented in Supplementary Table S2.

### 3.3. Risk of bias assessment

**Figure 2:**
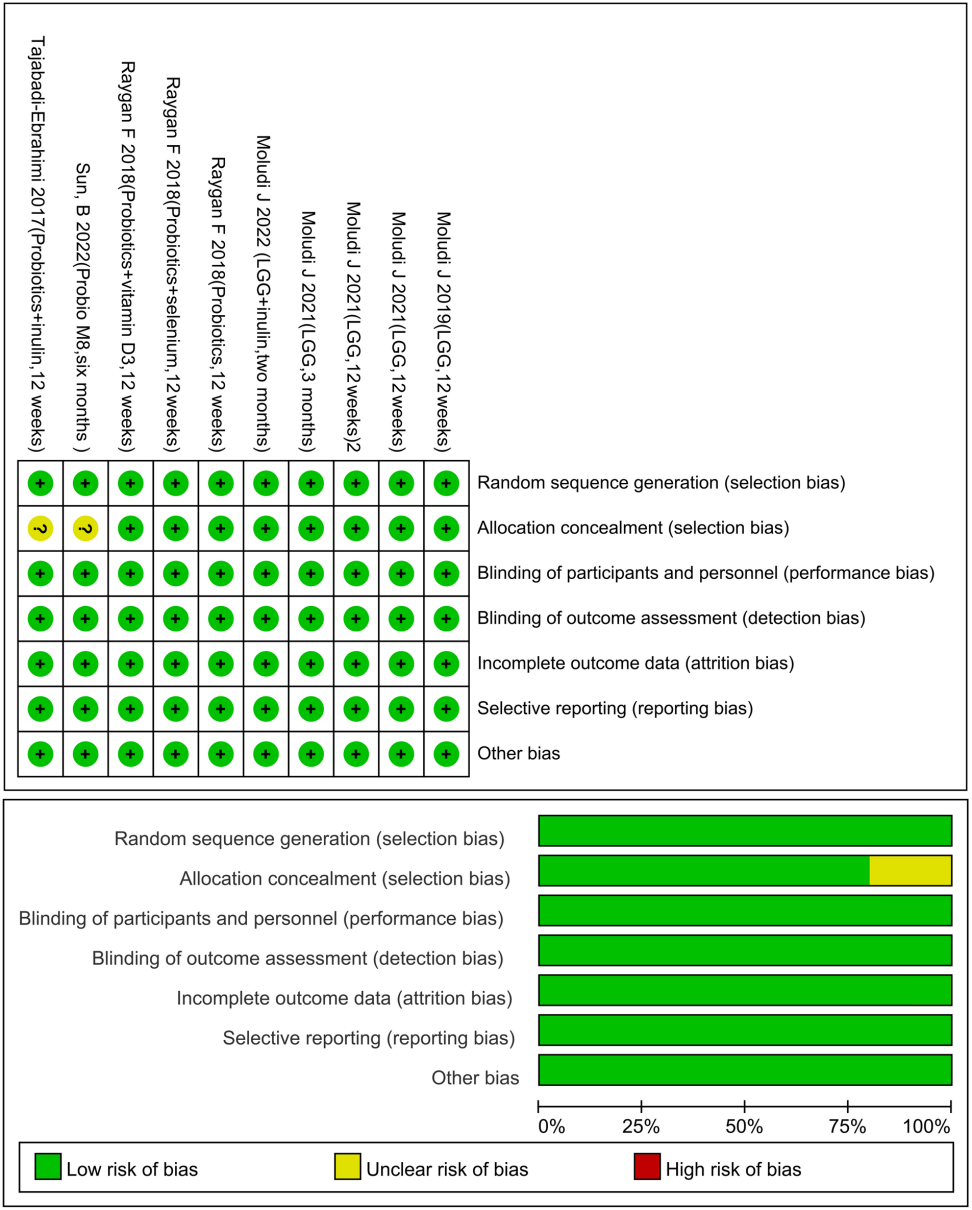
Risk of bias graph.

All articles were randomly assigned using computer software, and the records of the assignments were kept confidential by a third physician. The assignments were made using a double-masked method, and the data were considered reliable if the patient follow-up process and the number of missed visits were recorded in detail in the article. There must be drug company or institution funds in order to select results (Figure 2).

### 3.4. Lipid metabolism

Seven RCTs reported LDL-C data that could be included in the meta-analysis (5–7, 9–12). The result of the heterogeneity analysis was *I*^2^ = 28.0% and *P* = 0.21, indicating low heterogeneity among the seven studies. Therefore, the fixed-effects model was used. The final result indicates that probiotics may decrease LDL-C [WMD −9.13 (−13.17, −5.09), *P *< 0.01].

Six RCTs reported HDL-C, TC, and TG data that could be included in the meta-analysis (5–7, 9–11). The result of the heterogeneity analysis was *I*^2^ = 10.0% and *P* = 0.35 for HDL-C, the result of the heterogeneity analysis was *I*^2^ = 31.0% and *P* = 0.20 for TC, the result of the heterogeneity analysis was *I*^2^ = 0.0% and *P* = 0.72 for TG, indicating low heterogeneity among the six studies. Therefore, the fixed-effects model was used. The final result showed that probiotics may increase HDL-C [WMD 1.94 (0.32, 3.57), *P* = 0.02]. However, there was no statistically significant difference between the experimental group and the control group for TC [WMD −7.74 (−15.40, −0.07), *P* = 0.05] and TG [WMD −13.41 (−28.03, 1.21), *P* = 0.07], indicating low heterogeneity among the four studies. Therefore, the fixed-effects model was used. The results of the meta-analysis showed no statistically significant difference between the experimental group and the control group [WMD −2.83 (−6.34, 0.68), *P* = 0.11].

All four RCTs reported VLDL data that could be included in the meta-analysis (9, 13–15). The result of the heterogeneity analysis was *I*^2^ = 0.0% and *P* = 0.74, indicating low heterogeneity among the four studies. Therefore, the fixed-effects model was used. The results of the meta-analysis showed no statistically significant difference between the experimental group and the control group [WMD −2.83 (−6.34, 0.68), *P* = 0.11].

**Figure 3:**
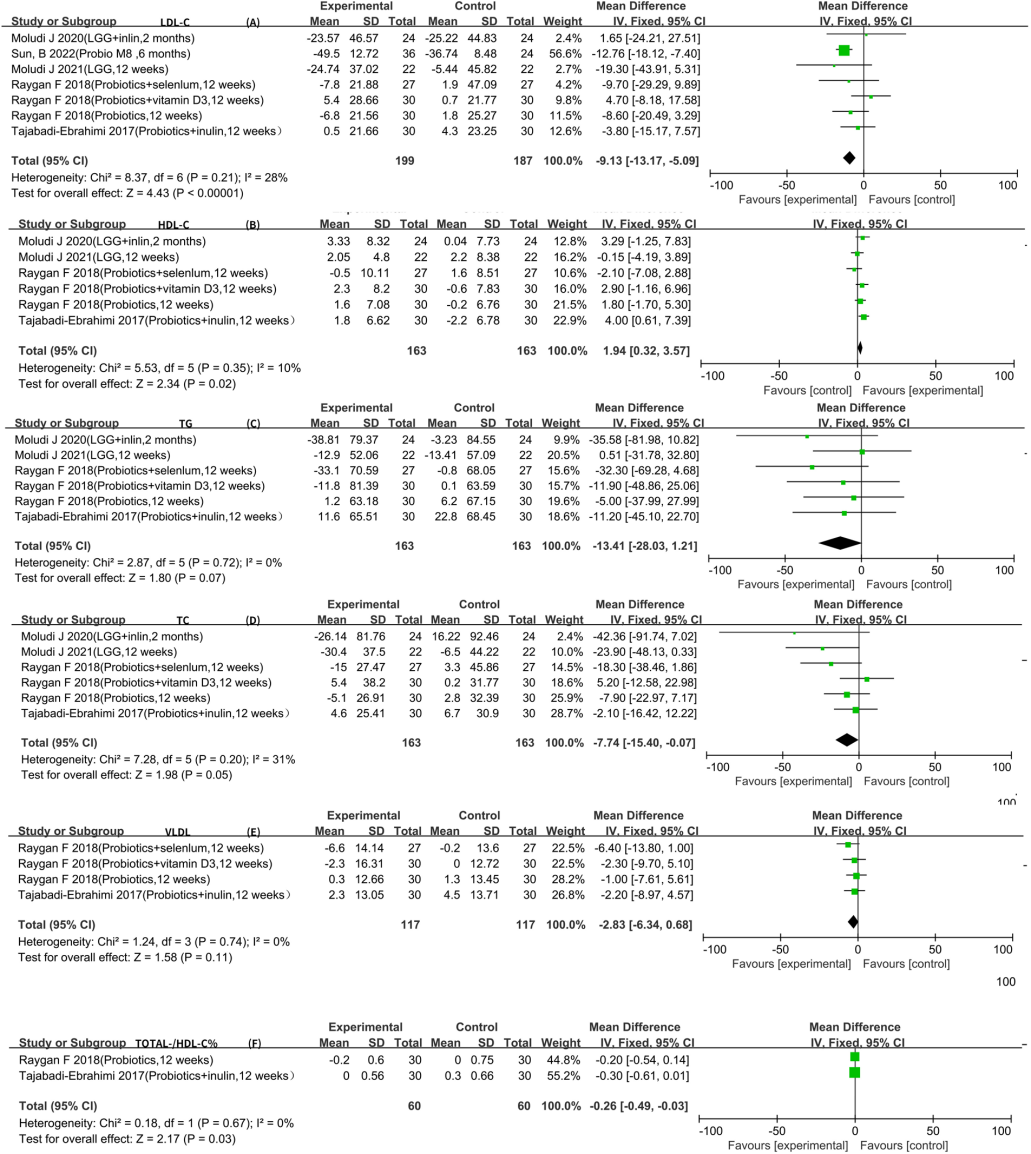
Result of lipid metabolism.

Two RCTs reported total-/HDL-cholesterol ratio (TOTAL-/HDL-C %) data that could be included in the meta-analysis (5, 9). The heterogeneity among the two studies was low, so the fixed-effects model was used. The final result showed that probiotics may decrease TOTAL-/HDL-C % [WMD −0.26 (−0.49, −0.03), *P* = 0.03] (Figure 3).

### 3.5. Glycometabolism

Six RCTs reported FPG data that could be included in the meta-analysis (5–7, 9–11). The result of the heterogeneity analysis was *I*^2^ = 1.1% and *P *= 0.41, indicating low heterogeneity among the six studies. Therefore, the fixed-effects model was used. The final result showed that probiotics may decrease FPG [WMD −13.60 (−23.57, −3.62), *P *< 0.01].

**Figure 4:**
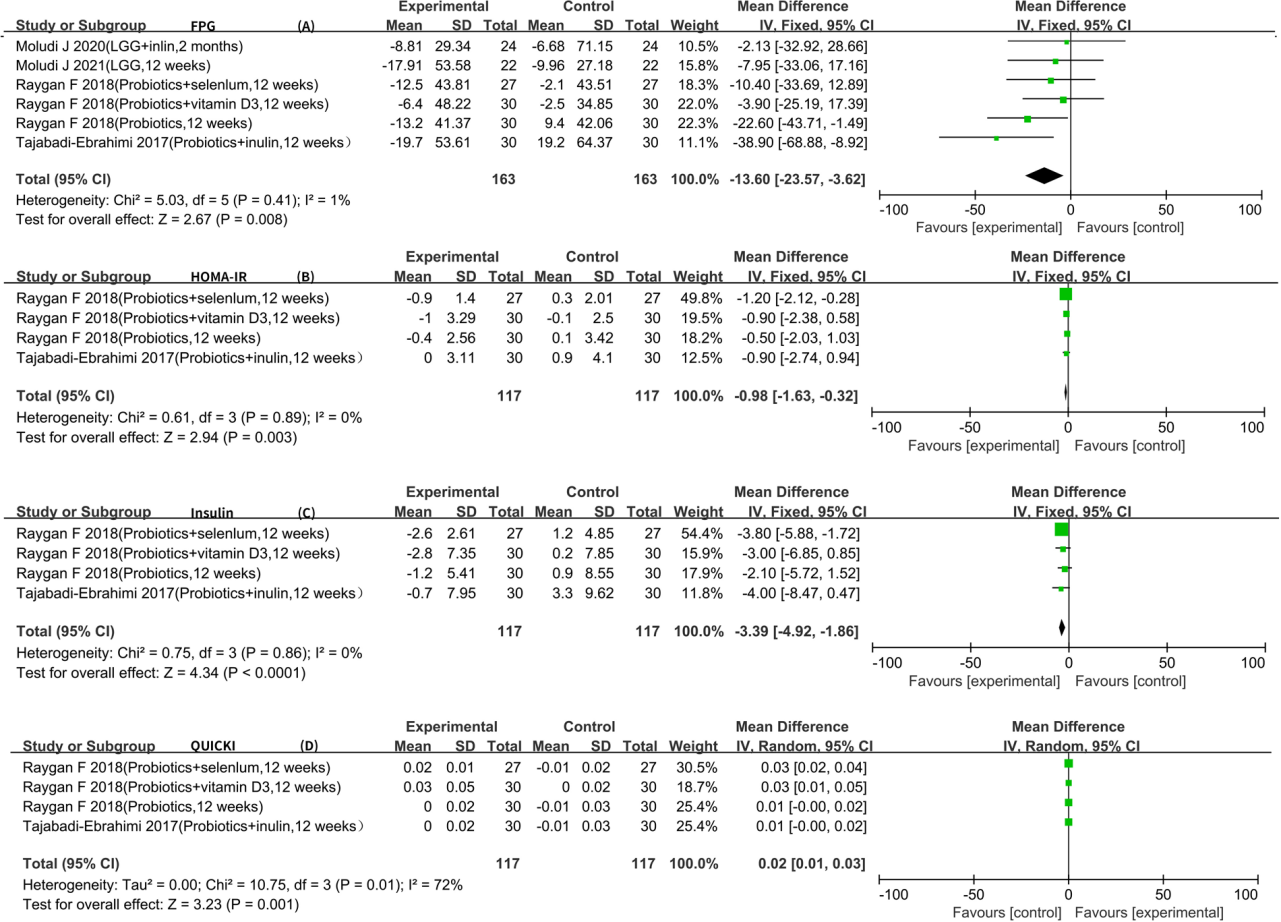
Result of glycometabolism.

Four RCTs reported insulin, HOMA-IR, and QUICKI data that could be included in the meta-analysis (5–7, 9). The result of the heterogeneity analysis was *I*^2^ = 0.0% and *P* = 0.86 for insulin, indicating low heterogeneity among the four studies. Therefore, the fixed-effects model was used. The results of the meta-analysis showed a statistically significant difference between the experimental group and the control group (*P *< 0.01), indicating that probiotics may decrease insulin [WMD −3.39 (−4.92, −1.86)]. The result of the heterogeneity analysis was *I*^2^ = 0.0% and *P* = 0.89 for HOMA-IR, indicating low heterogeneity among the four studies. Therefore, the fixed-effects model was used. The final result showed that probiotics may decrease HOMA-IR [WMD −0.98 (−1.63, −0.32), *P *< 0.01]. The result of the heterogeneity analysis was *I*^2^ = 72.0% and *P* = 0.01 for QUICKI, indicating high heterogeneity among the four studies. Therefore, the random-effects model was used. The results of the meta-analysis showed a statistically significant difference between the experimental group and the control group [WMD 0.02 (0.01, 0.03), *P *< 0.01] (Figure 4).

### 3.6. Blood pressure

**Figure 5:**
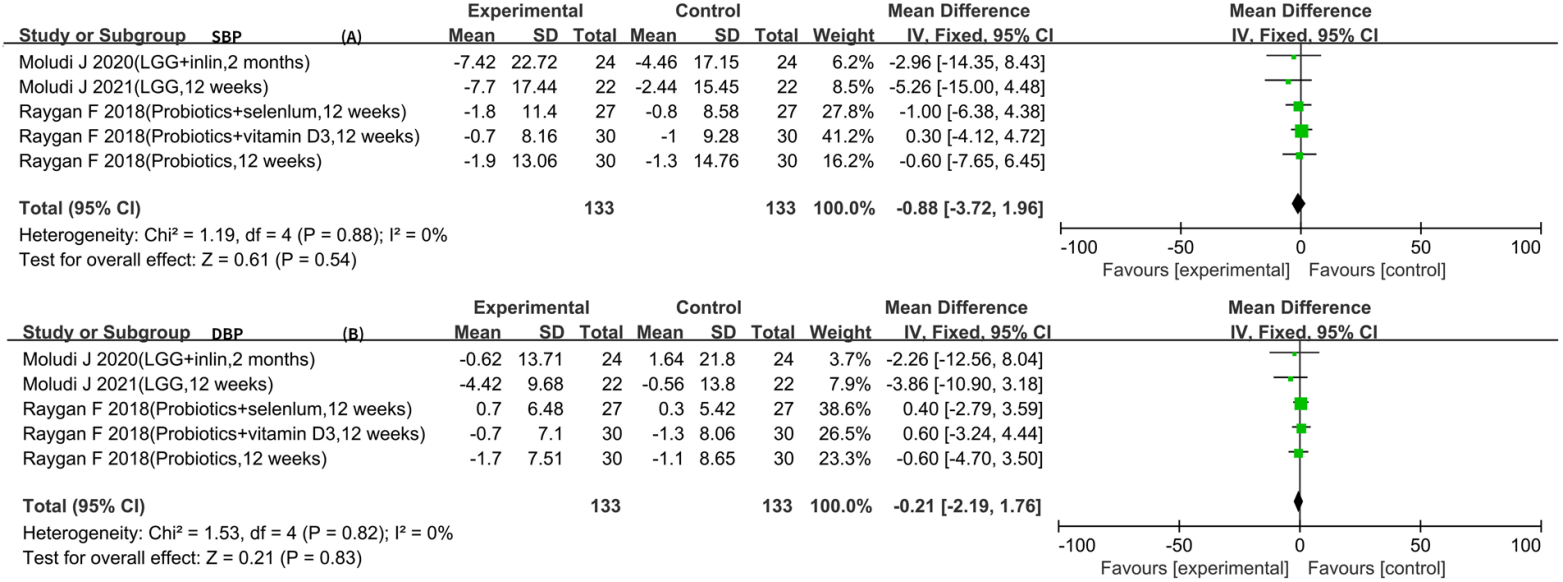
Result of blood pressure.

Five RCTs reported SBP and DBP data (5–7, 10, 11). The result of the heterogeneity analysis was *I*^2^ = 0.0% and *P* = 0.88 for SBP. The result of the heterogeneity analysis was *I*^2^ = 0.0% and *P* = 0.82 for DBP. These results indicate low heterogeneity among the five studies, so the fixed-effects model was used. The results of the meta-analysis showed that there was no statistically significant difference between the experimental group and the control group in terms of SBP [WMD −0.88 (−3.72, 1.96), *P* = 0.54] and DBP [WMD −0.21 (−2.19, 1.76), *P* = 0.83] (Figure 5).

### 3.7. Other risk factors

**Figure 6:**
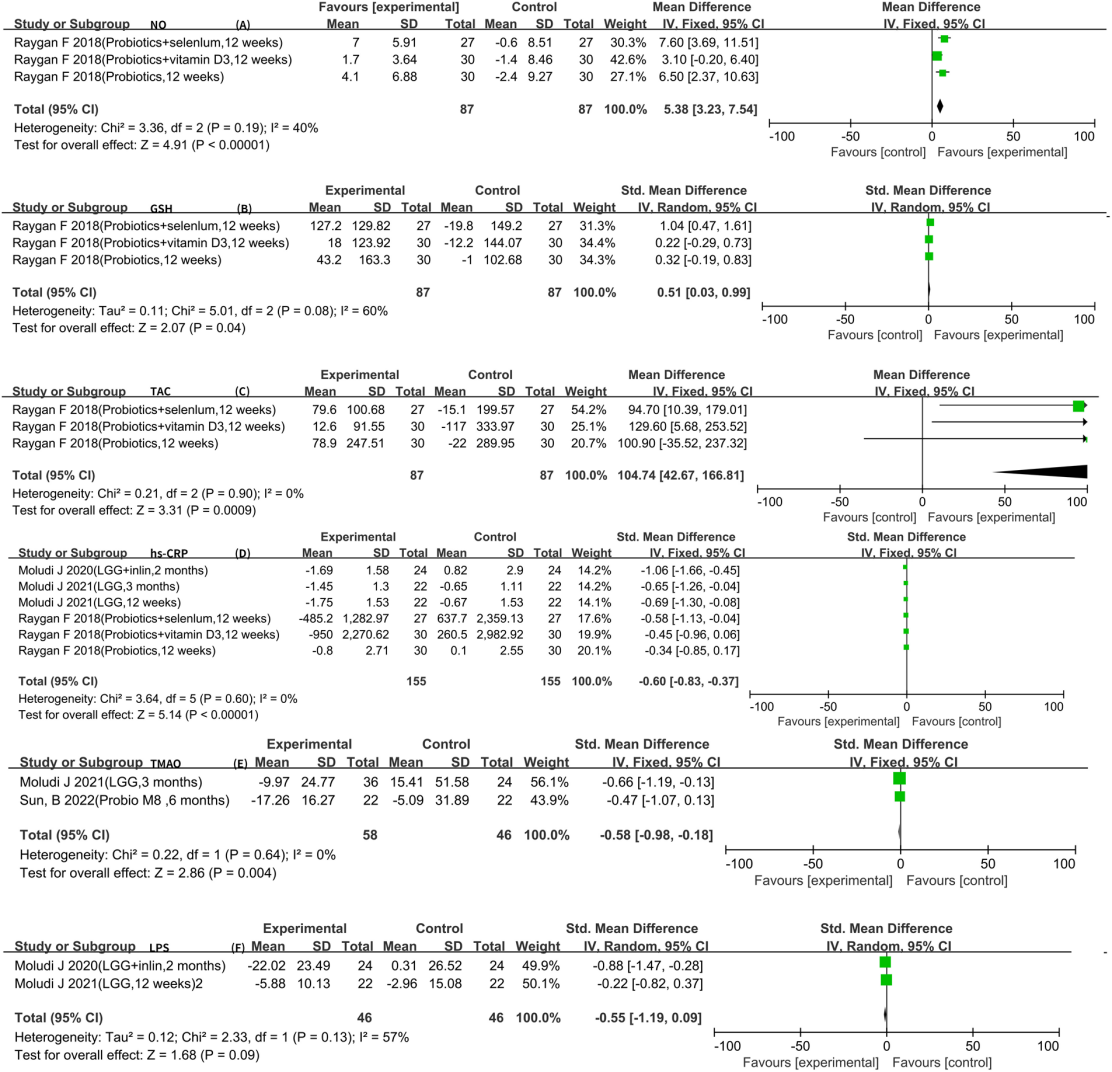
Result of other risk factors.

The analysis of the included literature revealed LPS, hs-CRP, GSH, TAC, and NO data that could be meta-analyzed. There were two articles for LPS (10, 11, 16). The result of the heterogeneity analysis was *I*^2^ = 57.0% and *P* = 0.13, indicating high heterogeneity between the two studies. Therefore, the random-effects model was used, which indicated that probiotics did not reach statistical significance in relation to LPS [SMD −0.55 (−1.19, 0.09), *P* = 0.09]. In addition, three RCTs reported GSH, TAC, and NO data (5–7), and six RCTs reported hs-CRP data that could be meta-analyzed (5–7, 11, 17, 18). In conclusion, using the fixed-effects model, it was observed that probiotics could further increase the levels of GSH [SMD 0.51 (0.03, 0.99), *P* = 0.04] and TAC [WMD 104.74 (42.67, 166.81), *P *< 0.01]. In the meantime, using a fixed-effects model, it was confirmed that probiotics could further increase the levels of NO [WMD 5.38 (3.23, 7.54), *P *< 0.01]. Six RCTs reported the hs-CPR data that can be meta-analyzed. The result of heterogeneity analysis was *I*^2^ = 0.0% and *P* = 0.60, which showed that the heterogeneity among the six studies was low, so the fixed-effects model was used. The results of the meta-analysis showed that there was a statistical difference between the experimental group and the control group (*P *< 0.01), which indicates that probiotics may decrease hs-CPR [SMD −0.60 (−0.83, −0.37)]. Furthermore, a meta-analysis of TMAO was conducted using a fixed-effects model, and it was observed that probiotics could further decrease the levels of TMAO [SMD −0.58 (−0.98, −0.18), *P *< 0.01] (Figure 6).

### 3.8. Adverse events

Moludi et al. found that 4.5% of the placebo group and 9% of the probiotic group reported symptoms, including gastrointestinal problems and gastric upset, after 12 weeks of intervention using *Lactobacillus rhamnosus* (18). However, Sun et al. did not find evidence that probiotics could increase the probability of adverse events (12). Other literature either did not report any associated adverse events or did not provide documentation regarding them.

## 4. Discussion

CAD, a cardiovascular disease characterized by a chronic inflammatory response and plaque accumulation, has become a major cause of cardiovascular death. While CAD drugs have shown promising effects in delaying coronary artery lesions, the mortality rate associated with CAD remains high. Therefore, it is crucial to further reduce the risk factors of coronary artery lesions (2). In recent years, intestinal microorganisms have gained attention in research on coronary artery lesions. The imbalance of intestinal microorganisms is closely linked to the progression of the disease. The advancements in microbial research have led to the proposal of the “gut–heart axis” theory, highlighting the close relationship between the intestine and coronary artery lesions. Intestinal disorders can contribute to the progression of coronary artery lesions (19). Gut microbes have the ability to positively modulate the host immune system, play an immunomodulatory role, defend against pathogenic microbes, and maintain normal physiological functions (20). Studies have indicated that treatment targeting gut microbes can delay the progression of coronary lesions (21). Although there have been meta-analyses on the effects of probiotics on lipid metabolism, glucose metabolism, blood pressure, and inflammatory factors (22, 23), they do not consider the effects of conventional drugs on the intestinal flora (24–26). Therefore, the objective of this meta-analysis was to examine the effect of probiotics on the risk factors for coronary artery lesions in conjunction with conventional drug therapy for CAD.

Regarding the effect of probiotics on lipid metabolism, although some studies have shown that probiotics can reduce TG and TC concentrations (24); this phenomenon was not observed in this meta-analysis. This discrepancy may be attributed to the specific population and conditions included in the analysis. Most of the existing meta-analyses do not restrict the disease population. In contrast, certain coronary drugs, such as aspirin (27) and atorvastatin (28), have been found to modulate the intestinal flora, which might diminish the impact of probiotics on the intestinal flora. Furthermore, the absence of a standardized dietary structure among patients is a crucial factor influencing the outcomes of intestinal flora changes. The included literature did not provide means to achieve dietary uniformity, which could be an important factor influencing the intervention outcomes. Therefore, considering that diabetic patients typically adhere to a diabetic diet, we conducted a subgroup analysis of patients with coronary heart disease combined with diabetes and different types of interventions and discovered a significant hypoglycemic effect of probiotics/synbiotics in this subgroup (Supplementary Tables S4, S6). However, such an effect was not observed in patients with CAD alone. In terms of intervention duration, time is another factor that affects the results. Significant reduction of LDL may require probiotic intervention for more than 12 weeks to have a notable effect, while hs-CRP shows a statistically significant difference with intervention lasting ≤12 weeks (Supplementary Table S5). This discrepancy may be related to the cycle of changes in the intestinal flora structure. In addition, probiotics can potentially increase the concentrations of TAC, GSH, and NO, which contribute to cardiovascular dilation and antioxidant capacity (29). Moreover, they can further decrease the levels of LPS, TMAO, and inflammatory response, all of which are beneficial in delaying the progression of coronary artery lesions and improving the prognosis of patients (30).

**Figure 7:**
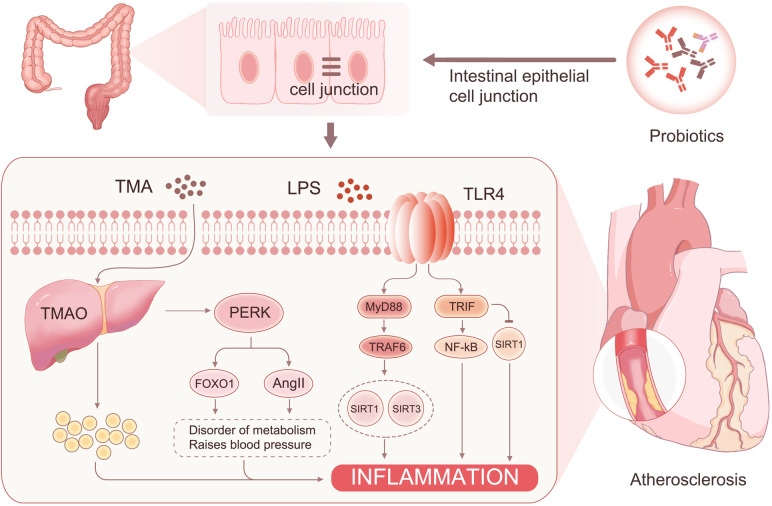
Probiotics reduce the risk factors for coronary artery disease.

The mechanism behind this phenomenon may originate from the following sources: (1) The intestinal flora reduces the permeability of intestinal epithelial cells through LRP6 and Wnt/β-catenin pathway activation (31). This reversal of intestinal barrier damage further decreases the entry of harmful substances such as LPS and TMAO into the bloodstream (32). (2) LPS can activate NF-κB through MyD88 and TRIF pathways, leading to the production of inflammatory factors and an increase in reactive oxygen species (33). This process ultimately sustains a chronic inflammatory response. TMAO binds to PERK (34), leading to increased FOXO1 activity and activation of the AKT signaling pathway, resulting in insulin resistance and diabetes. FOXO1 can also affect the farnesol X receptor and small heterodimeric chaperone receptor activation, which downregulates the expression of the *Cyp7a1* gene and upregulates the expression of bile acid transporter genes *Abcb11* and *Slc10a1* (35). These changes contribute to metabolic syndrome in patients. In addition, PERK can activate AngII, which leads to hypertension (36). However, probiotics reduce the entry of TMAO and LPS into the blood, thereby mitigating the effects of these risk factors. (3) GSH and NO play essential roles in the cardiovascular system. Studies have shown that GSH is negatively correlated with fasting blood glucose (37), and individuals with reduced plasma GSH levels have a significantly increased risk of cardiovascular disease (38). The increase in GSH and NO may be associated with a reduction in the metabolic syndrome of the patient (39). The elevated GSH levels can react with various free radicals, such as hydroxyl, hypochlorous, superoxide, and peroxynitrite (40, 41), and reduce hydrogen peroxide production, thereby mitigating cellular damage and oxidative stress (42). In addition, NO inhibits leukocyte adhesion to blood vessels and platelet aggregation and adhesion, contributing to the delay of coronary lesion progression (43) (Figure 7).

Therefore, this meta-analysis confirmed the effect of probiotics or synbiotics on the risk factors for coronary artery lesions in the treatment setting with conventional medications. After a rigorous literature screening process, 10 papers with 10 studies were finally included for data consolidation to objectively evaluate the effect of probiotics on coronary artery lesions. The results of this study showed that the addition of probiotics or synbiotics to conventional medications for CAD reduced the levels of LDL-C [WMD −9.13 (−13.17, −5.09)], FPG [WMD −13.60 (−23.57, −3.62)], insulin [WMD −3.39 (−4.92, −1.86)], LPS [SMD −0.55 (−1.19, −0.09)], hs-CRP [SMD −0.60 (−0.83, −0.37)], and TMAO [SMD −0.58 (−0.98, −0.18)] and increased the levels of HDL-C [WMD 1.94 (0.32, 3.57)] and NO [WMD 5.38 (3.23, 7.54)] but did not affect TG [WMD −13.41 (−28.03, 1.21)], SBP [WMD −0.88 (−3.72, 1.96)], and DBP [WMD −0.21 (−2.19, 1.76)].

This study has some limitations and strengths. The limitations include the following: (1) Since no specific drugs for coronary heart disease were given in the included literature, this may introduce bias to the intervention results. (2) The literature included in this study mainly consists of small-sample RCTs, with a maximum sample size of 36 cases in the trial group and 36 cases in the control group. The small sample size of some outcome indicators reduces the precision of the results. (3) Due to the limited number of included literature, the direct analysis of patient survival could not be conducted in the article. Instead, the risk factors for the prognosis of coronary heart disease were selected for analysis. Future studies should focus on direct analysis of major cardiovascular adverse events. (4) Most of the articles come from Iran, and the difference in ethnicity may have varying effects on the intervention effect. Future studies should consider a more diverse population. (5) The dietary structure and calorie intake of the subjects in different studies were not entirely consistent, which may influence the composition of the intestinal flora and interfere with the intervention results. (6) The number and lack of uniformity of probiotic species in the article hindered further subgroup analysis based on specific probiotic species.

Despite these limitations, the study also has strengths. This article demonstrated, through an evidence-based medical approach, that the use of probiotics/synbiotics in addition to conventional medications for coronary heart disease can further reduce the risk factors in patients, potentially improving their prognosis. Different diseases are closely related to the structure of intestinal flora, and most current articles do not separately analyze specific diseases. This study specifically conducted a meta-analysis of probiotic interventions to minimize the influence of disease on intervention factors. In this study, the control group consisted of patients receiving conventional drugs for coronary heart disease, while the experimental group received conventional drugs for coronary heart disease in combination with probiotic treatment. The study highlights the additional benefits provided by the addition of probiotics and suggests that future treatment regimens incorporating probiotics may further enhance the prognosis of patients with coronary heart disease.

In summary, the current evidence suggests that probiotics can significantly reduce the risk of coronary artery lesions, and the addition of probiotics to conventional medications for CAD may improve the prognosis of patients with CAD. However, the aforementioned findings still require further confirmation through large-sample and high-quality RCTs due to the potential impact of probiotic species type, intervention duration, CAD subtype, and sample size.

## Conclusion

Using probiotics or synbiotics, along with conventional medications for CAD, can further reduce the risk factors for coronary artery lesions and improve the prognosis of patients.

## Data Availability

The data sets presented in this study can be found in online repositories. The names of the repository/repositories and accession number(s) can be found in the article/**Supplementary Material**.

## References

[1] Akhabue E Thiboutot J Cheng JW Vittorio TJ Christodoulidis G Grady KM New and emerging risk factors for coronary heart disease. Am J Med Sci. (2014) 347(2):151–8. 10.1097/MAJ.0b013e31828aab45 23644407

[2] Tsao CW Aday AW Almarzooq ZI Alonso A Beaton AZ Bittencourt MS Heart disease and stroke statistics-2022 update: A report from the American Heart Association. Circulation. (2022) 145(8):e153–e639. 10.1161/CIR.0000000000001052 35078371

[3] Anhê FF Jensen B Perazza LR Tchernof A Schertzer JD Marette A Bacterial postbiotics as promising tools to mitigate cardiometabolic diseases. J Lipid Atheroscler. (2021) 10(2):123–9. 10.12997/jla.2021.10.2.123 34095007 PMC8159759

[4] Witkowski M Weeks TL Hazen SL. Gut microbiota and cardiovascular disease. Circ Res. (2020) 127(4):553–70. 10.1161/CIRCRESAHA.120.316242 32762536 PMC7416843

[5] Raygan F Rezavandi Z Bahmani F Ostadmohammadi V Mansournia MA Tajabadi-Ebrahimi M The effects of probiotic supplementation on metabolic status in type 2 diabetic patients with coronary heart disease. Diabetol Metab Syndr. (2018) 10:51. 10.1186/s13098-018-0353-2 29946368 PMC6008939

[6] Raygan F Ostadmohammadi V Bahmani F Asemi Z The effects of vitamin D and probiotic co-supplementation on mental health parameters and metabolic status in type 2 diabetic patients with coronary heart disease: a randomized, double-blind, placebo-controlled trial. Prog Neuropsychopharmacol Biol Psychiatry. (2018) 84(Pt A):50–5. 10.1016/j.pnpbp.2018.02.007 29432877

[7] Raygan F Ostadmohammadi V Asemi Z. The effects of probiotic and selenium co-supplementation on mental health parameters and metabolic profiles in type 2 diabetic patients with coronary heart disease: a randomized, double-blind, placebo-controlled trial. Clin Nutr. (2019) 38(4):1594–8. 10.1016/j.clnu.2018.07.017 30057015

[8] Bock PM Telo GH Ramalho R Sbaraini M Leivas G Martins AF The effect of probiotics, prebiotics or synbiotics on metabolic outcomes in individuals with diabetes: a systematic review and meta-analysis. Diabetologia. (2021) 64(1):26–41. 10.1007/s00125-020-05295-1 33047170

[9] Tajabadi-Ebrahimi M Sharifi N Farrokhian A Raygan F Karamali F Razzaghi R A randomized controlled clinical trial investigating the effect of synbiotic administration on markers of insulin metabolism and lipid profiles in overweight type 2 diabetic patients with coronary heart disease. Exp Clin Endocrinol Diabetes. (2017) 125(1):21–7. 10.1055/s-0042-105441 27219886

[10] Moludi J Alizadeh M Behrooz M Maleki V Seyed MM Golmohammadi A Interactive effect of probiotics supplementation and weight loss diet on metabolic syndrome features in patients with coronary artery diseases: a double-blind, placebo-controlled, randomized clinical trial. Am J Lifestyle Med. (2021) 15(6):653–63. 10.1177/1559827619843833 34916886 PMC8669900

[11] Moludi J Khedmatgozar H Nachvak SM Abdollahzad H Moradinazar M Sadeghpour TA The effects of co-administration of probiotics and prebiotics on chronic inflammation, and depression symptoms in patients with coronary artery diseases: a randomized clinical trial. Nutr Neurosci. (2022) 25(8):1659–68. 10.1080/1028415X.2021.1889451 33641656

[12] Sun B Ma T Li Y Yang N Li B Zhou X *Bifidobacterium lactis* probio-M8 adjuvant treatment confers added benefits to patients with coronary artery disease via target modulation of the gut-heart/-brain axes. mSystems. (2022) 7(2):e10022. 10.1128/msystems.00100-22 PMC9040731 35343796

[13] Raygan F Ostadmohammadi V Bahmani F Asemi Z The effects of vitamin D and probiotic co-supplementation on mental health parameters and metabolic status in type 2 diabetic patients with coronary heart disease: a randomized, double-blind, placebo-controlled trial. Prog Neuropsychopharmacol Biol Psychiatry. (2018) 84(Pt A):50–5. 10.1016/j.pnpbp.2018.02.007 29432877

[14] Raygan F Rezavandi Z Bahmani F Ostadmohammadi V Mansournia MA Tajabadi-Ebrahimi M The effects of probiotic supplementation on metabolic status in type 2 diabetic patients with coronary heart disease IRCT2017082733941N5 IRCT. Diabetol Metab Syndr. (2018) 10:51. 10.1186/s13098-018-0353-2 29946368 PMC6008939

[15] Raygan F Ostadmohammadi V Asemi Z. The effects of probiotic and selenium co-supplementation on mental health parameters and metabolic profiles in type 2 diabetic patients with coronary heart disease: a randomized, double-blind, placebo-controlled trial. Clin Nutr. (2019) 38(4):1594–8. 10.1016/j.clnu.2018.07.017 30057015

[16] Moludi J Kafil HS Qaisar SA Gholizadeh P Alizadeh M Vayghyan HJ Effect of probiotic supplementation along with calorie restriction on metabolic endotoxemia, and inflammation markers in coronary artery disease patients: a double blind placebo controlled randomized clinical trial. Nutr J. (2021) 20(1):47. 10.1186/s12937-021-00703-7 34074289 PMC8170788

[17] Moludi J Saiedi S Ebrahimi B Alizadeh M Khajebishak Y Ghadimi SS Probiotics supplementation on cardiac remodeling following myocardial infarction: a single-center double-blind clinical study. J Cardiovasc Transl Res. (2021) 14(2):299–307. 10.1007/s12265-020-10052-1 32681453

[18] Moludi J Alizadeh M Mohammadzad M Davari M The effect of probiotic supplementation on depressive symptoms and quality of life in patients after myocardial infarction: results of a preliminary double-blind clinical trial. Psychosom Med. (2019) 81(9):770–7. 10.1097/PSY.0000000000000749 31592939

[19] Zhang Y Zhang X Chen D Lu J Gong Q Fang J Causal associations between gut microbiome and cardiovascular disease: a Mendelian randomization study. Front Cardiovasc Med. (2022) 9:971376. 10.3389/fcvm.2022.971376 36110421 PMC9470126

[20] Suriano F Nyström E Sergi D Gustafsson JK Diet, microbiota, and the mucus layer: the guardians of our health. Front Immunol. (2022) 13:953196. 10.3389/fimmu.2022.953196 36177011 PMC9513540

[21] Xie B Zu X Wang Z Xu X Liu G Liu R Ginsenoside Rc ameliorated atherosclerosis via regulating gut microbiota and fecal metabolites. Front Pharmacol. (2022) 13:990476. 10.3389/fphar.2022.990476 36188559 PMC9520581

[22] Pontes K Guedes MR Cunha M Mattos SS Barreto SM Neves MF Effects of probiotics on body adiposity and cardiovascular risk markers in individuals with overweight and obesity: a systematic review and meta-analysis of randomized controlled trials. Clin Nutr. (2021) 40(8):4915–31. 10.1016/j.clnu.2021.06.023 34358838

[23] Zheng HJ Guo J Wang Q Wang L Wang Y Zhang F Probiotics, prebiotics, and synbiotics for the improvement of metabolic profiles in patients with chronic kidney disease: a systematic review and meta-analysis of randomized controlled trials. Crit Rev Food Sci Nutr. (2021) 61(4):577–98. 10.1080/10408398.2020.1740645 32329633

[24] Liang T Xie X Wu L Li L Yang L Gao H Comparative analysis of the efficacies of probiotic supplementation and glucose-lowering drugs for the treatment of type 2 diabetes: a systematic review and meta-analysis. Front Nutr. (2022) 9:825897. 10.3389/fnut.2022.825897 35923194 PMC9339904

[25] Hadi A Pourmasoumi M Kazemi M Najafgholizadeh A Marx W Efficacy of synbiotic interventions on blood pressure: a systematic review and meta-analysis of clinical trials. Crit Rev Food Sci Nutr. (2022) 62(20):5582–91. 10.1080/10408398.2021.1888278 33612008

[26] Guo YT Peng YC Yen HY Wu JC Hou WH Effects of probiotic supplementation on immune and inflammatory markers in athletes: a meta-analysis of randomized clinical trials. Medicina (Kaunas). (2022) 58(9):1188. 10.3390/medicina58091188 36143865 PMC9505795

[27] Chi T Zhao Q Wang P. Fecal 16S rRNA gene sequencing analysis of changes in the gut microbiota of rats with low-dose aspirin-related intestinal injury. Biomed Res Int. (2021) 2021:8848686. 10.1155/2021/8848686 33954200 PMC8060078

[28] Li DY Li XS Chaikijurajai T Relation of statin use to gut microbial trimethylamine N-oxide and cardiovascular risk. Am J Cardiol. (2022) 178:26–34. 10.1016/j.amjcard.2022.05.010 35787338 PMC13317319

[29] Li DY Li XS Chaikijurajai T Li L Wang Z Hazen SL Xinshuaining preparation protects H9c2 cells from H(2)O(2)-induced oxidative damage through the PI3K/Akt/Nrf-2 signaling pathway. Clin Exp Hypertens. (2022):1–9. 10.1080/10641963.2022.2131806. [Epub ahead of print]. 36266998

[30] Khosravi M Poursaleh A Ghasempour G Farhad S Najafi M The effects of oxidative stress on the development of atherosclerosis. Biol Chem. (2019) 400(6):711–32. 10.1515/hsz-2018-0397 30864421

[31] Izadparast F Riahi-Zajani B Yarmohammadi F Hayes AW Karimi G Protective effect of berberine against LPS-induced injury in the intestine: a review. Cell Cycle. (2022) 21(22):2365–78. 10.1080/15384101.2022.2100682 35852392 PMC9645259

[32] Choroszy M Sobieszczańska B Litwinowicz K Łaczmański Ł Chmielarz M Walczuk U Co-toxicity of endotoxin and indoxyl sulfate, gut-derived bacterial metabolites, to vascular endothelial cells in coronary arterial disease accompanied by gut dysbiosis. Nutrients. (2022) 14(3):424. 10.3390/nu14030424 35276782 PMC8840142

[33] Sheng J Xu J. Association of coronary artery disease with toll-like receptor 4 genetic variants: a meta-analysis. Adv Clin Exp Med. (2019) 28(5):651–8. 10.17219/acem/91791 30784241

[34] Chen S Henderson A Petriello MC Romano KA Gearing M Miao J Trimethylamine N-oxide binds and activates PERK to promote metabolic dysfunction. Cell Metab. (2019) 30(6):1141–51. 10.1016/j.cmet.2019.08.021 31543404

[35] Li Y Zhang L Ren P Yang Y Li S Qin X Qing-Xue-Xiao-Zhi formula attenuates atherosclerosis by inhibiting macrophage lipid accumulation and inflammatory response via TLR4/MyD88/NF-κB pathway regulation. Phytomedicine. (2021) 93:153812. 10.1016/j.phymed.2021.153812 34753029

[36] Hu J Xu J Shen S Zhang W Chen H Sun X Trimethylamine N-oxide promotes abdominal aortic aneurysm formation by aggravating aortic smooth muscle cell senescence in mice. J Cardiovasc Transl Res. (2022) 15(5):1064–74. 10.1007/s12265-022-10211-6 35143032 PMC9622512

[37] Karolczak K Kubalczyk P Głowacki R Pietruszyński R Watała C An inverse relationship between plasma glutathione concentration and fasting glycemia in patients with coronary artery disease and concomitant type 2 diabetes: a pilot study. Adv Clin Exp Med. (2017) 26(9):1359–66. 10.17219/acem/65441 29442456

[38] Gould RL Pazdro R. Impact of supplementary amino acids, micronutrients, and overall diet on glutathione homeostasis. Nutrients. (2019) 11(5):1056. 10.3390/nu11051056 31083508 PMC6566166

[39] Belizário JE Faintuch J Garay-Malpartida M. Gut microbiome dysbiosis and immunometabolism: new frontiers for treatment of metabolic diseases. Mediators Inflamm. (2018) 2018:2037838. 10.1155/2018/2037838 30622429 PMC6304917

[40] Matuz-Mares D Riveros-Rosas H Vilchis-Landeros MM Vázquez-Meza H Glutathione participation in the prevention of cardiovascular diseases. Antioxidants (Basel). (2021) 10(8):1220. 10.3390/antiox10081220 34439468 PMC8389000

[41] Wu G Fang YZ Yang S Lupton JR Turner ND Glutathione metabolism and its implications for health. J Nutr. (2004) 134(3):489–92. 10.1093/jn/134.3.489 14988435

[42] Lu SC. Regulation of glutathione synthesis. Mol Aspects Med. (2009) 30(1–2):42–59. 10.1016/j.mam.2008.05.005 18601945 PMC2704241

[43] Cohen KE Katunaric B Schulz ME SenthilKumar G Young MS Mace JE Role of adiponectin receptor 1 in promoting nitric oxide-mediated flow-induced dilation in the human microvasculature. Front Pharmacol. (2022) 13:875900. 10.3389/fphar.2022.875900 35444544 PMC9014203

