# Supplementary material for: Meta-analysis of the effect of probiotics or synbiotics on the risk factors in patients with coronary artery disease
Source: Front Cardiovasc Med. 2023 Aug 2;10:1154888. doi: 10.3389/fcvm.2023.1154888 (PMC10436219; doi:10.3389/fcvm.2023.1154888)
Supplement: Supplementary file 1 [file Table1.docx]

Supplementary Table S1: Search strategy

| Database | Search Strategy |
| --- | --- |
| PubMed (755) | Search: (((((((((((((Coronary Artery Disease[MeSH Terms]) OR (Left Main Coronary Artery Disease[Title/Abstract])) OR (Left Main Disease[Title/Abstract])) OR (Left Main Diseases[Title/Abstract])) OR (Left Main Coronary Disease[Title/Abstract])) OR (Coronary Arteriosclerosis[Title/Abstract])) OR (Atherosclerosis[Title/Abstract])) OR (Coronary Atherosclerosis[Title/Abstract])) OR (coronary disease[Title/Abstract])) OR (coronary artery disease[Title/Abstract])) OR (cardiovascular events[Title/Abstract])) OR (angina pectoris[Title/Abstract])) OR ((((((((((((((Myocardial Infarction[MeSH Terms]) OR (Infarction, Myocardial[MeSH Terms])) OR (Infarctions, Myocardial[MeSH Terms])) OR (Myocardial Infarctions[MeSH Terms])) OR (Cardiovascular Stroke[MeSH Terms])) OR (Cardiovascular Strokes[MeSH Terms])) OR (Stroke, Cardiovascular[MeSH Terms])) OR (Strokes, Cardiovascular[MeSH Terms])) OR (Myocardial Infarct[MeSH Terms])) OR (Infarct, Myocardial[MeSH Terms])) OR (Infarcts, Myocardial[MeSH Terms])) OR (Myocardial Infarcts[MeSH Terms])) OR (Heart Attack[MeSH Terms])) OR (Heart Attacks[MeSH Terms]))) AND (((((Probiotics[MeSH Terms]) OR ((((((Probiotics[Title/Abstract]) OR (Probiotic[Title/Abstract])) OR (probiotic agents[Title/Abstract])) OR (live bacteria[Title/Abstract])) OR (beneficial bacteria[Title/Abstract])) OR (beneficial microorganisms[Title/Abstract]))) OR (((Prebiotics[MeSH Terms]) OR (Prebiotic[Title/Abstract])) OR (Prebiotics[Title/Abstract]))) OR (((((Synbiotics[MeSH Terms]) OR (Synbiotics[Title/Abstract])) OR (Synbiotic[Title/Abstract])) OR (Symbiotic[Title/Abstract])) OR (Symbiotics[Title/Abstract]))) OR (((gastrointestinal microbiome[MeSH Terms]) OR (gut microbiome[Title/Abstract])) OR (gut flora[Title/Abstract]))) Sort by: Most Recent |
| Scopus (1187) | ( ( ( TITLE-ABS-KEY ( coronary AND artery AND disease ) OR TITLE-ABS-KEY ( left AND main AND coronary AND artery AND disease ) OR TITLE-ABS-KEY ( left AND main AND disease ) OR TITLE-ABS-KEY ( left AND main AND coronary AND disease ) OR TITLE-ABS-KEY ( coronary AND arteriosclerosis ) OR TITLE-ABS-KEY ( atherosclerosis ) OR TITLE-ABS-KEY ( coronary AND atherosclerosis ) OR TITLE-ABS-KEY ( coronary AND disease ) OR TITLE-ABS-KEY ( coronary AND artery AND disease ) OR TITLE-ABS-KEY ( cardiovascular AND events ) OR TITLE-ABS-KEY ( angina AND pectoris ) OR TITLE-ABS-KEY ( myocardial AND infarction ) OR TITLE-ABS-KEY ( infarction, AND myocardial ) OR TITLE-ABS-KEY ( infarctions, AND myocardial ) OR TITLE-ABS-KEY ( myocardial AND infarctions ) OR TITLE-ABS-KEY ( cardiovascular AND stroke ) OR TITLE-ABS-KEY ( cardiovascular AND strokes ) OR TITLE-ABS-KEY ( stroke, AND cardiovascular ) OR TITLE-ABS-KEY ( strokes, AND cardiovascular ) OR TITLE-ABS-KEY ( myocardial AND infarct ) OR TITLE-ABS-KEY ( infarct, AND myocardial ) OR TITLE-ABS-KEY ( infarcts, AND myocardial ) OR TITLE-ABS-KEY ( myocardial AND infarcts ) OR TITLE-ABS-KEY ( heart AND attack ) OR TITLE-ABS-KEY ( heart AND attacks ) ) ) AND ( ( TITLE-ABS-KEY ( probiotics ) OR TITLE-ABS-KEY ( probiotics ) OR TITLE-ABS-KEY ( probiotic ) OR TITLE-ABS-KEY ( probiotic AND agents ) OR TITLE-ABS-KEY ( live AND bacteria ) OR TITLE-ABS-KEY ( beneficial AND bacteria ) OR TITLE-ABS-KEY ( beneficial AND microorganisms ) OR TITLE-ABS-KEY ( prebiotics ) OR TITLE-ABS-KEY ( prebiotic ) OR TITLE-ABS-KEY ( prebiotics ) OR TITLE-ABS-KEY ( synbiotics ) OR TITLE-ABS-KEY ( synbiotic ) OR TITLE-ABS-KEY ( symbiotic ) OR TITLE-ABS-KEY ( symbiotics ) OR TITLE-ABS-KEY ( gastrointestinal AND microbiome ) OR TITLE-ABS-KEY ( gut AND microbiome ) OR TITLE-ABS-KEY ( gut AND flora ) ) ) ) AND ( ( TITLE-ABS-KEY ( clinical AND trials ) OR TITLE-ABS-KEY ( clinical AND trials AND as AND a AND topic ) OR TITLE-ABS-KEY ( randomized AND controlled AND trial ) OR TITLE-ABS-KEY ( randomized AND controlled AND trials AND as AND topic ) OR TITLE-ABS-KEY ( controlled AND clinical AND trial ) OR TITLE-ABS-KEY ( controlled AND clinical AND trials ) OR TITLE-ABS-KEY ( random AND allocation ) OR TITLE-ABS-KEY ( double-blind AND method ) OR TITLE-ABS-KEY ( single-blind AND method ) OR TITLE-ABS-KEY ( cross-over AND studies ) OR TITLE-ABS-KEY ( placebos ) OR TITLE-ABS-KEY ( multicenter AND study ) OR TITLE-ABS-KEY ( double AND blind AND procedure ) OR TITLE-ABS-KEY ( single AND blind AND procedure ) OR TITLE-ABS-KEY ( crossover AND procedure ) OR TITLE-ABS-KEY ( clinical AND trial ) OR TITLE-ABS-KEY ( controlled AND study ) OR TITLE-ABS-KEY ( randomization ) OR TITLE-ABS-KEY ( placebo ) OR TITLE-ABS-KEY ( factorial AND design ) OR TITLE-ABS-KEY ( factorial AND trial ) OR TITLE-ABS-KEY ( clinical ) OR TITLE-ABS-KEY ( trial ) OR TITLE-ABS-KEY ( rct ) OR TITLE-ABS-KEY ( random ) OR TITLE-ABS-KEY ( blind ) ) ) |
| Web of Science (1427) | #1:((((((TS=(Probiotics)) OR TS=(Probiotics)) OR TS=(Probiotic)) OR TS=(probiotic agents)) OR TS=(live bacteria)) OR TS=(beneficial bacteria)) OR TS=(beneficial microorganisms) OR (((((((TS=(Prebiotics)) OR TS=(Prebiotic)) OR TS=(Prebiotics)) OR TS=(Synbiotics)) OR TS=(Synbiotics)) OR TS=(Synbiotic)) OR TS=(Symbiotic)) OR TS=(Symbiotics) OR ((TS=(gastrointestinal microbiome)) OR TS=(gut microbiome)) OR TS=(gut flora)  #2:((((((((((((((((((((((((TS=(Coronary Artery Disease)) OR TS=(Left Main Coronary Artery Disease)) OR TS=(Left Main Disease)) OR TS=(Left Main Coronary Disease)) OR TS=(Coronary Arteriosclerosis)) OR TS=(Atherosclerosis )) OR TS=(Coronary Atherosclerosis)) OR TS=(coronary disease)) OR TS=(coronary artery disease)) OR TS=(cardiovascular events)) OR TS=(angina pectoris)) OR TS=(Myocardial Infarction)) OR TS=(Infarction, Myocardial)) OR TS=(Infarctions, Myocardial)) OR TS=(Myocardial Infarctions)) OR TS=(Cardiovascular Stroke)) OR TS=(Cardiovascular Strokes)) OR TS=(Stroke, Cardiovascular)) OR TS=(Strokes, Cardiovascular)) OR TS=(Myocardial Infarct)) OR TS=(Infarct, Myocardial)) OR TS=(Infarcts, Myocardial)) OR TS=(Myocardial Infarcts)) OR TS=(Heart Attack)) OR TS=(Heart Attacks)  #1 AND #2 |
| Embase (3437) | #1:'left main coronary artery disease':ti,ab,kw OR 'left main disease':ti,ab,kw OR 'left main coronary disease':ti,ab,kw OR 'coronary arteriosclerosis':ti,ab,kw OR 'beneficial bacteria':ti,ab,kw OR 'beneficial microorganisms':ti,ab,kw OR atherosclerosis:ti,ab,kw OR 'coronary atherosclerosis':ti,ab,kw OR 'coronary disease':ti,ab,kw OR 'coronary artery disease':ti,ab,kw OR 'cardiovascular events':ti,ab,kw OR 'angina pectoris':ti,ab,kw OR 'myocardial infarction':ti,ab,kw OR 'infarction, myocardial':ti,ab,kw OR 'infarctions, myocardial':ti,ab,kw OR 'myocardial infarctions':ti,ab,kw OR 'cardiovascular stroke':ti,ab,kw OR 'cardiovascular strokes':ti,ab,kw OR 'stroke, cardiovascular':ti,ab,kw OR 'strokes, cardiovascular':ti,ab,kw OR 'myocardial infarct':ti,ab,kw OR 'infarct, myocardial':ti,ab,kw OR 'infarcts, myocardial':ti,ab,kw OR 'myocardial infarcts':ti,ab,kw OR 'heart attack':ti,ab,kw OR 'heart attacks':ti,ab,kw  #2:probiotics:ti,ab,kw OR probiotic:ti,ab,kw OR 'probiotic agents':ti,ab,kw OR 'live bacteria':ti,ab,kw OR 'beneficial bacteria':ti,ab,kw OR 'beneficial microorganisms':ti,ab,kw OR prebiotic:ti,ab,kw OR prebiotics:ti,ab,kw OR synbiotics:ti,ab,kw OR synbiotic:ti,ab,kw OR symbiotic:ti,ab,kw OR symbiotics:ti,ab,kw OR 'gastrointestinal microbiome':ti,ab,kw OR 'gut microbiome':ti,ab,kw OR 'gut flora':ti,ab,kw  #1 AND #2 |
| Cochrane (193) | #1 (Coronary Artery Disease):ti,ab,kw OR (Left Main Coronary Artery Disease):ti,ab,kw OR (Left Main Disease):ti,ab,kw OR (Left Main Coronary Disease):ti,ab,kw OR (Coronary Arteriosclerosis):ti,ab,kw OR (Atherosclerosis):ti,ab,kw OR (Coronary Atherosclerosis):ti,ab,kw OR (coronary disease):ti,ab,kw OR (coronary artery disease):ti,ab,kw OR (cardiovascular events):ti,ab,kw OR (angina pectoris):ti,ab,kw OR (Myocardial Infarction):ti,ab,kw OR (Infarction, Myocardial):ti,ab,kw OR (Infarctions, Myocardial):ti,ab,kw OR (Myocardial Infarctions):ti,ab,kw OR (Cardiovascular Stroke):ti,ab,kw OR (Cardiovascular Strokes):ti,ab,kw OR (Stroke, Cardiovascular):ti,ab,kw OR (Strokes, Cardiovascular):ti,ab,kw OR (Myocardial Infarct):ti,ab,kw OR (Infarct, Myocardial):ti,ab,kw OR (Infarcts, Myocardial):ti,ab,kw OR (Myocardial Infarcts):ti,ab,kw OR (Heart Attack):ti,ab,kw OR (Heart Attacks):ti,ab,kw  #2 (Probiotics):ti,ab,kw OR (Probiotics):ti,ab,kw OR (Probiotic):ti,ab,kw OR (probiotic agents):ti,ab,kw OR (live bacteria):ti,ab,kw OR (beneficial bacteria):ti,ab,kw OR (beneficial microorganisms):ti,ab,kw OR (Prebiotics):ti,ab,kw OR (Prebiotic):ti,ab,kw OR (Prebiotics):ti,ab,kw OR (Synbiotics):ti,ab,kw OR (Synbiotics):ti,ab,kw OR (Synbiotic):ti,ab,kw OR (Symbiotic):ti,ab,kw OR (Symbiotics):ti,ab,kw OR (gastrointestinal microbiome):ti,ab,kw OR (gut microbiome):ti,ab,kw OR (gut flora):ti,ab,kw  #1 AND #2 |
