# Supplementary material for: Meta-analysis of the effect of probiotics or synbiotics on the risk factors in patients with coronary artery disease
Source: Front Cardiovasc Med. 2023 Aug 2;10:1154888. doi: 10.3389/fcvm.2023.1154888 (PMC10436219; doi:10.3389/fcvm.2023.1154888)
Supplement: Supplementary file 2 [file Table2.docx]

| **Disease** | **Year** | **Study** | **Trial/Control** | | **Intervention** | **Duration** | **indicators** |
| --- | --- | --- | --- | --- | --- | --- | --- |
| CAD | 2022 | Sun B^[15]^ | | 24/36 | *Probio-M8,* 3×10^10^CFU/day | 6 months | A; M; |
| CAD | 2022 | Moludi J^[14]^ | | 24/24 | *Lactobacillus rhamnosus,* 1.9×10^9^CFU/day and 15 g inulin | 2 months | A; B; C; D; G; K; L; N; R; |
| CAD | 2021 | Moludi J^[13]^ | | 22/22 | *Lactobacillus rhamnosus,* 1.9×10^9^CFU/day | 12 weeks | R; |
| MI | 2021 | Moludi J^[12]^ | | 22/22 | *Lactobacillus rhamnosus,* 1.9×10^9^CFU/day | 3 months | N; M; |
| CAD | 2021 | Moludi J^[11]^ | | 22/22 | *Lactobacillus rhamnosus,* 1.9×10^9^CFU/day | 12 weeks | A; B; C; D; G; K; L; |
| CAD | 2019 | Moludi J^[7]^ | | 22/22 | *Lactobacillus rhamnosus,* 1.9×10^9^CFU/day | 12 weeks | N; |
| T2DM and CAD | 2018 | Raygan, F^[10]^ | | 27/27 | *Lactobacillus acidophilus; Lactobacillus reuteri; Lactoba-cillus fermentum; Bifidobacterium bifidum,* (each 2×10^9^CFU/day) | 12 weeks | A; B; C; D; E; G; H; I; J; K; L; N; O; P; Q; |
| T2DM and CAD | 2018 | Raygan F^[6]^ | | 30/30 | *Lactobacillus acidophilus; Bifidobacterium bifidum; Lactobacillus reuteri; Lactobacillus fermentum,* (each 2×10^9^CFU/day) and vitamin D 350,000 IU/2 weeks | 12 weeks | A; B; C; D; E; G; H; I; J; K; L; N; O; P; Q; |
| T2DM and CAD | 2018 | Raygan, F^[5]^ | | 30/30 | *Bifidobacterium bifidum; Lactobacillus casei;*  *Lactobacillus acidophilus,* (each 2×10^9^CFU/day) | 12 weeks | A; B; C; D; E; F; G; H; I; J; K; L; N; O; P; Q; |
| T2DM and CAD | 2017 | Tajabadi-Ebrahimi M^[9]^ | | 30/30 | *Lactobacillus acidophilus; Lactobacillus casei; Bifidobacterium bifidum,* (each 2×10^9^CFU/day) and 800 mg inulin | 12 weeks | A; B; C; D; E; F; G; H; I; J; |

Supplementary Table S2: A: LDL-C; B: HDL-C; C: TC; D: TG; E: VLDL; F: TOTAL-/HDL-C%; G: FPG; H: Insulin; I: HOMA-IR; J: QUICKI; K: SBP; L: DBP; M: TMAO; N: hs-CRP; O: GSH; P: NO; Q: TAC; R: LPS;
