# Supplementary material for: Meta-analysis of the effect of probiotics or synbiotics on the risk factors in patients with coronary artery disease
Source: Front Cardiovasc Med. 2023 Aug 2;10:1154888. doi: 10.3389/fcvm.2023.1154888 (PMC10436219; doi:10.3389/fcvm.2023.1154888)
Supplement: Supplementary file 3 [file Table3.docx]

| **Quality assessment** | | | | | | | | **No of patients** | | **Effect** | **Quality** | **Importance** |
| --- | --- | --- | --- | --- | --- | --- | --- | --- | --- | --- | --- | --- |
| **Outcome** | **No of studies** | **Design** | **Risk of bias** | **Inconsistency** | **Indirectness** | **Imprecision** | **Other considerations** | **experiment** | **Control** | **Relative (95% CI)** |  |  |
| LDL-C | 7 | randomised trials | no serious risk of bias | serious | no serious indirectness | no serious imprecision | none | 199 | 187 | MD 9.13 lower (13.17 to 5.09 lower) | ÅÅÅO MODERATE | IMPORTANT |
| HDL-C | 6 | randomised trials | no serious risk of bias | serious | no serious indirectness | no serious imprecision | none | 163 | 163 | MD 1.94 higher (0.32 to 3.57 higher) | ÅÅÅO MODERATE | IMPORTANT |
| TG | 6 | randomised trials | no serious risk of bias | serious | no serious indirectness | serious | none | 163 | 163 | MD 13.41 lower (28.03 lower to 1.21 higher) | ÅÅOO LOW | IMPORTANT |
| TC | 6 | randomised trials | no serious risk of bias | serious | no serious indirectness | serious | none | 163 | 163 | MD 7.74 lower (15.4 to 0.07 lower) | ÅÅOO LOW | IMPORTANT |
| VLDL | 4 | randomised trials | no serious risk of bias | serious | no serious indirectness | no serious imprecision | none | 117 | 117 | MD 2.83 lower (6.34 lower to 0.68 higher) | ÅÅÅO MODERATE | IMPORTANT |
| TOTAL/HDL-C% | 2 | randomised trials | no serious risk of bias | serious | no serious indirectness | no serious imprecision | none | 60 | 60 | MD 0.26 lower (0.49 to 0.03 lower) | ÅÅÅO MODERATE | IMPORTANT |
| FPG | 6 | randomised trials | no serious risk of bias | serious | no serious indirectness | serious | none | 163 | 163 | MD 13.6 lower (23.57 to 3.62 lower) | ÅÅOO LOW | IMPORTANT |
| HOMA-IR | 4 | randomised trials | no serious risk of bias | serious | no serious indirectness | no serious imprecision | none | 117 | 117 | MD 0.98 lower (1.63 to 0.32 lower) | ÅÅÅO MODERATE | IMPORTANT |
| Insulin | 4 | randomised trials | no serious risk of bias | serious | no serious indirectness | no serious imprecision | none | 117 | 117 | MD 3.39 lower (4.92 to 1.86 lower) | ÅÅÅO MODERATE | IMPORTANT |
| QUICKI | 4 | randomised trials | no serious risk of bias | serious | no serious indirectness | no serious imprecision | none | 117 | 117 | MD 0.02 higher (0.01 to 0.03 higher) | ÅÅÅO MODERATE | IMPORTANT |
| SBP | 5 | randomised trials | no serious risk of bias | serious | no serious indirectness | no serious imprecision | none | 133 | 133 | MD 0.88 lower (3.72 lower to 1.96 higher) | ÅÅÅO MODERATE | IMPORTANT |
| DBP | 5 | randomised trials | no serious risk of bias | serious | no serious indirectness | no serious imprecision | none | 133 | 133 | MD 0.21 lower (2.19 lower to 1.76 higher) | ÅÅÅO MODERATE | IMPORTANT |
| NO | 3 | randomised trials | no serious risk of bias | serious | no serious indirectness | serious | none | 87 | 87 | MD 5.38 higher (3.23 to 7.54 higher) | ÅÅOO LOW | IMPORTANT |
| GSH | 3 | randomised trials | no serious risk of bias | serious | no serious indirectness | serious | none | 87 | 87 | SMD 0.51 higher (0.03 to 0.99 higher) | ÅÅOO LOW | IMPORTANT |
| TAC | 3 | randomised trials | no serious risk of bias | serious | no serious indirectness | serious | none | 87 | 87 | SMD 0.49 higher (0.19 to 0.79 higher) | ÅÅOO LOW | IMPORTANT |
| hs-CRP | 6 | randomised trials | no serious risk of bias | serious | no serious indirectness | no serious imprecision | none | 155 | 155 | SMD 0.6 higher (0.37 to 0.83 higher) | ÅÅÅO MODERATE | IMPORTANT |
| TMAO | 2 | randomised trials | no serious risk of bias | serious | no serious indirectness | serious | none | 58 | 46 | SMD 0.58 lower (0.98 to 0.18 lower) | ÅÅOO LOW | IMPORTANT |
| LPS | 2 | randomised trials | no serious risk of bias | serious | no serious indirectness | serious | none | 46 | 46 | SMD 0.55 lower (1.19 lower to 0.09 higher) | ÅÅOO LOW | IMPORTANT |

Supplementary Table S3
