# Supplementary material for: Meta-analysis of the effect of probiotics or synbiotics on the risk factors in patients with coronary artery disease
Source: Front Cardiovasc Med. 2023 Aug 2;10:1154888. doi: 10.3389/fcvm.2023.1154888 (PMC10436219; doi:10.3389/fcvm.2023.1154888)
Supplement: Supplementary file 4 [file Table4.docx]

| **Outcomes** | **Heterogeneity test** | | | **Model** | **Effect** | | |
| --- | --- | --- | --- | --- | --- | --- | --- |
|  | **No of studies** | ***I*^2^(%)** | ***p* value** |  | **WMD/SMD (95%CI)** | **Egger test**  **(p value)** | **p value** |
| **probiotics** | | | | | | | |
| LDL-C (mg/dL) | 3 | 0.0 | 0.70 | fixed | -12.33 (-17.13, -7.53) | 0.917 | ＜0.001 |
| HDL-C (mg/dL) | 2 | 0.0 | 0.47 | fixed | 0.96 (-1.68, 3.61) | _ | 0.476 |
| TG (mg/dL) | 2 | 0.0 | 0.81 | fixed | -2.18 (-25.26, 20.89) | _ | 0.853 |
| TC (mg/dL) | 2 | 17.2 | 0.27 | fixed | -12.36 ( -25.16, 0.43) | _ | 0.058 |
| FPG (mg/dL) | 2 | 0.0 | 0.38 | fixed | -16.53 (-32.69, -0.37) | _ | 0.045 |
| DBP (mmHg) | 2 | 0.0 | 0.43 | fixed | -1.42 (-4.97, 2.12) | _ | 0.431 |
| SBP (mmHg) | 2 | 0.0 | 0.45 | fixed | -2.20 (-7.91, 3.51) | _ | 0.449 |
| hs-CRP（SMD） | 3 | 0.0 | 0.89 | fixed | -0.91 (-1.42, -0.39) | 0.751 | ＜0.001 |
| TMAO（SMD） | 2 | 0.0 | 0.64 | fixed | -0.59 (-0.99, -0.19) | _ | 0.004 |
| **synbiotics** | | | | | | | |
| LDL-C (mg/dL) | 4 | 0.0 | 0.62 | fixed | -1.34 (-8.82, 6.14) | 0.852 | 0.726 |
| HDL-C (mg/dL) | 4 | 28.2 | 0.24 | fixed | 2.53 (0.48, 4.59) | 0.208 | 0.016 |
| TG (mg/dL) | 4 | 0.0 | 0.73 | fixed | -20.95 (-39.85, -2.04) | 0.293 | 0.030 |
| TC (mg/dL) | 4 | 43.3 | 0.15 | fixed | -5.15 (-14.72, 4.42) | 0.250 | 0.292 |
| VLDL (mg/dL) | 3 | 0.0 | 0.66 | fixed | -3.55 (-7.69, 0.59) | 0.637 | 0.093 |
| FPG (mg/dL) | 4 | 26.1 | 0.25 | fixed | -11.79 (-24.47, 0.89) | 0.504 | 0.068 |
| HOMA-IR | 3 | 0.0 | 0.92 | fixed | -1.08 (-1.80, -0.36) | 0.200 | 0.003 |
| Insulin (mIU/mL) | 3 | 0.0 | 0.93 | fixed | -3.67 (-5.37, -1.98) | 0.765 | ＜0.001 |
| QUICKI | 3 | 75.2 | 0.02 | random | 0.02 (0.01, 0.04) | 0.788 | 0.002 |
| DBP (mmHg) | 3 | 0.0 | 0.88 | fixed | 0.33 (-2.05, 2.72) | 0.226 | 0.784 |
| SBP (mmHg) | 3 | 0.0 | 0.84 | fixed | -0.45 (-3.72, 2.82) | 0.279 | 0.788 |
| NO (µmol/L) | 2 | 66.4 | 0.08 | random | 5.22 (0.82, 9.62) | _ | 0.020 |
| GSH（SMD） | 2 | 77.8 | 0.03 | random | 0.63 (-0.18, 1.44) | _ | 0.129 |
| TAC (mmol/L) | 2 | 0.0 | 0.65 | fixed | 105.74 (36.04, 175.45) | _ | 0.003 |
| hs-CRP（SMD） | 3 | 18.5 | 0.29 | fixed | -0.67 (-0.99, -0.35) | 0.094 | ＜0.001 |

Supplementary Table S4: Subgroup analysis by probiotic and synbiotic species
