# Supplementary material for: Meta-analysis of the effect of probiotics or synbiotics on the risk factors in patients with coronary artery disease
Source: Front Cardiovasc Med. 2023 Aug 2;10:1154888. doi: 10.3389/fcvm.2023.1154888 (PMC10436219; doi:10.3389/fcvm.2023.1154888)
Supplement: Supplementary file 5 [file Table5.docx]

| **Outcomes** | **Heterogeneity test** | | | **Model** | **Effect** | | |
| --- | --- | --- | --- | --- | --- | --- | --- |
|  | **No of studies** | ***I*^2^ (%)** | ***p* value** |  | **WMD/SMD (95%CI)** | **Egger test**  **(*p* value)** | ***p* value** |
| **≤12w** | | | | | | | |
| LDL-C (mg/dL) | 5 | 2.1 | 0.39 | fixed | -4.74 ( -11.06, 1.57) | 0.313 | 0.141 |
| hs-CRP（SMD） | 4 | 0.0 | 0.82 | fixed | -0.51 (-0.78, -0.24) | 0.084 | ＜0.001 |
| **＞12w** | | | | | | | |
| LDL-C (mg/dL) | 2 | 12.6 | 0.28 | fixed | -12.17 (-17.42, -6.91) | _ | ＜0.001 |
| hs-CRP（SMD） | 2 | 79.9 | 0.03 | random | -1.19 (-1.81, -0.56) | _ | ＜0.001 |

Supplementary Table S5: Subgroup analysis by intervention time line
