# Supplementary material for: Meta-analysis of the effect of probiotics or synbiotics on the risk factors in patients with coronary artery disease
Source: Front Cardiovasc Med. 2023 Aug 2;10:1154888. doi: 10.3389/fcvm.2023.1154888 (PMC10436219; doi:10.3389/fcvm.2023.1154888)
Supplement: Supplementary file 6 [file Table6.docx]

| **Outcomes** | **Heterogeneity test** | | | **Model** | **Effect** | | |
| --- | --- | --- | --- | --- | --- | --- | --- |
|  | **No of studies** | ***I*^2^ (%)** | ***p* value** |  | **WMD/SMD (95%CI)** | **Egger test**  **(*p* value)** | ***p* value** |
| **CAD** | | | | | | | |
| LDL-C (mg/dL) | 3 | 0.0 | 0.484 | fixed | -12.48 (-17.61, -7.34) | 0.786 | ＜0.001 |
| HDL-C (mg/dL) | 2 | 18.8 | 0.267 | fixed | 1.37 (-1.65, 4.38) | _ | 0.375 |
| TG (mg/dL) | 2 | 36.1 | 0.211 | fixed | -11.26 (-37.77, 15.24) | _ | 0.405 |
| TC (mg/dL) | 2 | 0.0 | 0.511 | fixed | -27.48 (-49.23, -5.73) | _ | 0.013 |
| FPG (mg/dL) | 2 | 0.0 | 0.774 | fixed | -5.63 (-25.08, 13.83) | _ | 0.571 |
| DBP (mmHg) | 2 | 0.0 | 0.802 | fixed | -3.35 (-9.16, 2.46) | _ | 0.259 |
| SBP (mmHg) | 2 | 0.0 | 0.764 | fixed | -4.29 (-11.69, 3.11) | _ | 0.256 |
| hs-CRP（SMD） | 2 | 0.0 | 0.585 | fixed | -0.81 (-1.17, -0.46) | _ | ＜0.001 |
| TMAO（SMD） | 2 | 0.0 | 0.64 | fixed | -0.59 (-0.99, -0.19) | _ | 0.004 |
| **T2DM and CAD** | | | | | | | |
| LDL-C (mg/dL) | 4 | 0.0 | 0.449 | fixed | -3.72 (-10.25, 2.81) | 0.778 | 0.264 |
| HDL-C (mg/dL) | 4 | 26.9 | 0.250 | fixed | 2.18 (0.25, 4.10) | 0.162 | 0.027 |
| TG (mg/dL) | 4 | 0.0 | 0.738 | fixed | -14.35 (-31.89, 3.19) | 0.270 | 0.109 |
| TC (mg/dL) | 4 | 7.3 | 0.357 | fixed | -4.94 (-13.13, 3.25) | 0.624 | 0.237 |
| VLDL (mg/dL) | 4 | 0.0 | 0.743 | fixed | -2.83 (-6.34, 0.68) | 0.283 | 0.114 |
| Total-/HDL—C% | 2 | 0.0 | 0.672 | fixed | -0.26 (-0.48, -0.03) | _ | 0.029 |
| FPG (mg/dL) | 4 | 26.4 | 0.254 | fixed | -16.44 (-28.06, -4.82) | 0.288 | 0.006 |
| HOMA-IR | 4 | 0.0 | 0.893 | fixed | -0.978 (-1.63, -0.32) | 0.234 | 0.003 |
| Insulin (mIU/mL) | 4 | 0.0 | 0.862 | fixed | -3.39 (-4.92, -1.86) | 0.536 | ＜0.001 |
| QUICKI | 4 | 76.5 | 0.005 | random | 0.02 (0.01, 0.03) | 0.601 | 0.002 |
| DBP (mmHg) | 3 | 0.0 | 0.904 | fixed | 0.19 (-1.91, 2.30) | 0.603 | 0.855 |
| SBP (mmHg) | 3 | 0.0 | 0.931 | fixed | -0.29 (-3.37, 2.78) | 0.554 | 0.851 |
| NO (µmol/L) | 3 | 40.5 | 0.186 | fixed | 5.38 (3.23, 7.54) | 0.281 | ＜0.001 |
| GSH（SMD） | 3 | 61.3 | 0.075 | random | 0.52 (0.03, 1.01) | 0.059 | 0.039 |
| TAC (mmol/L) | 3 | 0.0 | 0.899 | fixed | 104.74 (42.67, 166.81) | 0.579 | ＜0.001 |
| hs-CRP（SMD） | 3 | 0.0 | 0.807 | fixed | -0.46 (-0.76, -0.16) | 0.265 | 0.003 |

Supplementary Table S6 Subgroup analysis by presence or absence of comorbid diabetes mellitus
