# Supplementary material for: Meta-analysis of the effect of probiotics or synbiotics on the risk factors in patients with coronary artery disease
Source: Front Cardiovasc Med. 2023 Aug 2;10:1154888. doi: 10.3389/fcvm.2023.1154888 (PMC10436219; doi:10.3389/fcvm.2023.1154888)

Means and standard deviations of changes in outcome variables before and after the intervention were calculated according to the method provided in Cochrane Handbook 5.0.2 (16.1.3.2) with the formula $\mathrm{Mean}_{E, change}=\mathrm{Mean}_{E, final}-\mathrm{Mean}_{E, baseline}$_,_ ${SD}_{E, change}=\sqrt{{SD}_{E, baseline}^{2}+{SD}_{E, final}^{2}-(2*Corr*{SD}_{E, baseline}*{SD}_{E, final})}$, Corr = 0.50. Statistical heterogeneity of included studies was analyzed using the Q test and *I*^2^ test. *I*^2^ < 50% was considered low heterogeneity, and *I*^2^ > 50% was considered high heterogeneity.

| LDL | | | | | | | |
| --- | --- | --- | --- | --- | --- | --- | --- |
| Author | all | nc | tc | cfmean | cfsd | tfmean | tfsd |
| Moludi J 2020(LGG+inlin,2 months) | 48 | 24 | 24 | -25.22 | 44.83 | -23.57 | 46.57 |
| Sun, B 2022(Probio M8 ,6 months ) | 60 | 24 | 36 | -36.74 | 8.48 | -49.50 | 12.72 |
| Moludi J 2021(LGG,12weeks) | 44 | 22 | 22 | -5.44 | 45.82 | -24.74 | 37.02 |
| Raygan F 2018(Probiotics+selenlum,12 weeks) | 54 | 27 | 27 | 1.90 | 47.09 | -7.80 | 21.88 |
| Raygan F 2018(Probiotics+vitamin D3,12 weeks) | 60 | 30 | 30 | 0.70 | 21.77 | 5.40 | 28.66 |
| Raygan F 2018(Probiotics,12 weeks) | 60 | 30 | 30 | 1.80 | 25.27 | -6.80 | 21.56 |
| Tajabadi-Ebrahimi 2017(Probiotics +inulin,12 weeks) | 60 | 30 | 30 | 4.30 | 23.25 | 0.50 | 21.66 |

**CODE：metan tc tfmean tfsd nc cfmean cfsd, label(namevar=author) fixed nostandard**

**CODE：metafunnel _ES _seES**

**CODE：metabias6 _ES _seES, graph(egger)**

**CODE：metaninf tc tfmean tfsd nc cfmean cfsd, label(namevar=author) fixed nostandard**


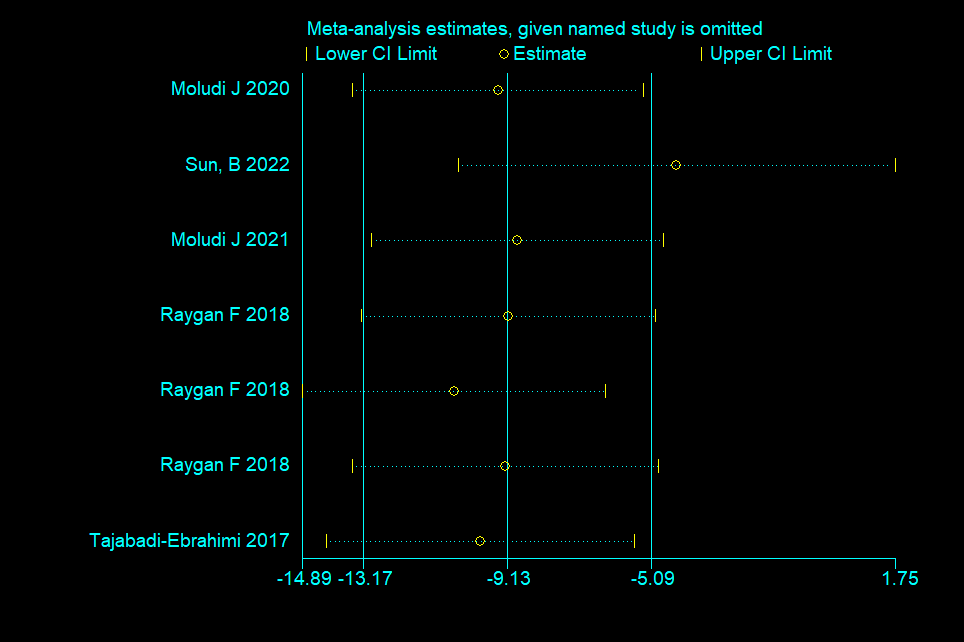


| HDL | | | | | | | |
| --- | --- | --- | --- | --- | --- | --- | --- |
| Author | all | nc | tc | cfmean | cfsd | tfmean | tfsd |
| Moludi J 2020(LGG+inlin,2 months) | 48 | 24 | 24 | 0.04 | 7.73 | 3.33 | 8.32 |
| Moludi J 2021(LGG,12weeks) | 44 | 22 | 22 | 2.20 | 8.38 | 2.05 | 4.80 |
| Raygan F 2018(Probiotics+selenlum,12 weeks) | 54 | 27 | 27 | 1.60 | 8.51 | -0.50 | 10.11 |
| Raygan F 2018(Probiotics+vitamin D3,12 weeks) | 60 | 30 | 30 | -0.60 | 7.83 | 2.30 | 8.20 |
| Raygan F 2018(Probiotics,12 weeks) | 60 | 30 | 30 | -0.20 | 6.76 | 1.60 | 7.08 |
| Tajabadi-Ebrahimi 2017(Probiotics +inulin,12 weeks) | 60 | 30 | 30 | -2.20 | 6.78 | 1.80 | 6.62 |

**CODE：metan tc tfmean tfsd nc cfmean cfsd, label(namevar=author) fixed nostandard**

**CODE：metafunnel _ES _seES**


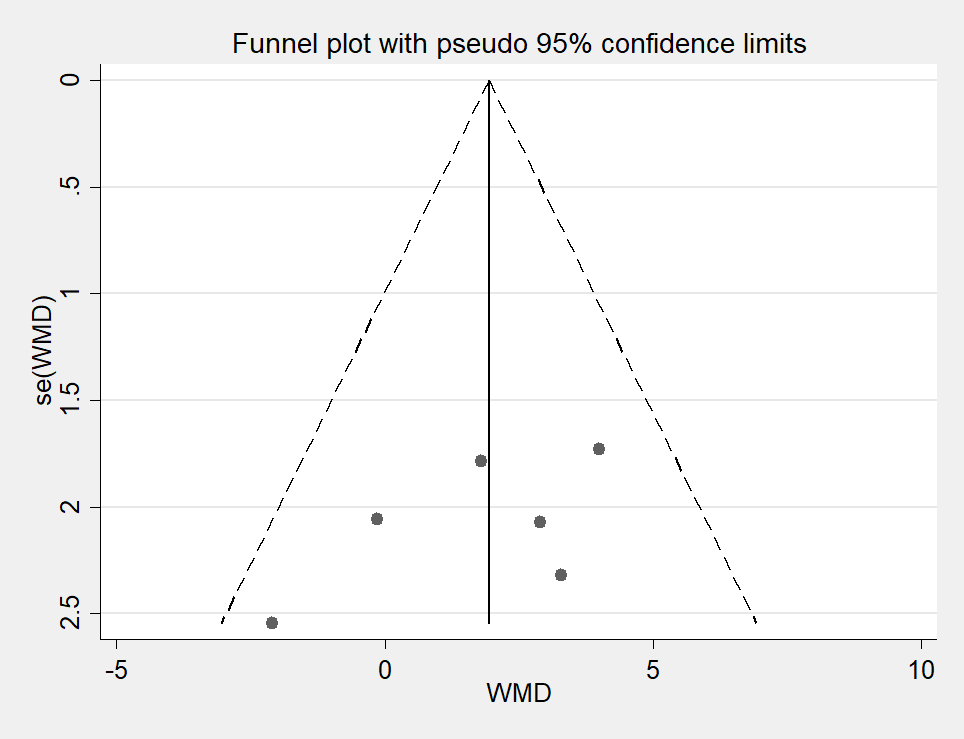


**CODE：metabias6 _ES _seES, graph(egger)**

**CODE：metaninf tc tfmean tfsd nc cfmean cfsd, label(namevar=author) fixed nostandard**


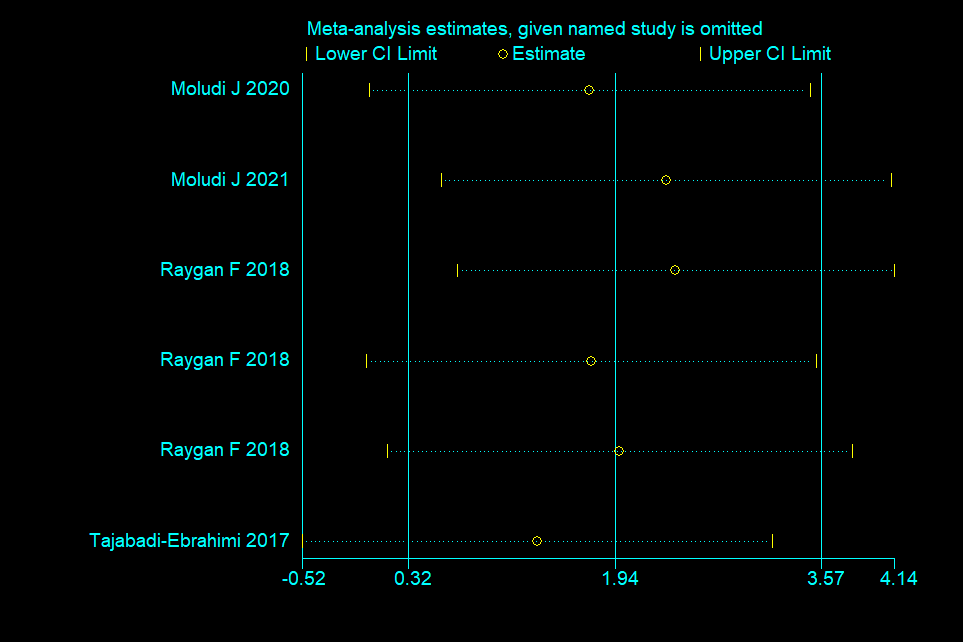


| TG | | | | | | | |
| --- | --- | --- | --- | --- | --- | --- | --- |
| Author | all | nc | tc | cfmean | cfsd | tfmean | tfsd |
| Moludi J 2020(LGG+inlin,2 months) | 48 | 24 | 24 | -3.23 | 84.55 | -38.81 | 79.37 |
| Moludi J 2021(LGG,12weeks) | 44 | 22 | 22 | -13.41 | 57.09 | -12.90 | 52.06 |
| Raygan F 2018(Probiotics+selenlum,12 weeks) | 54 | 27 | 27 | -0.80 | 68.05 | -33.10 | 70.59 |
| Raygan F 2018(Probiotics+vitamin D3,12 weeks) | 60 | 30 | 30 | 0.10 | 63.59 | -11.80 | 81.39 |
| Raygan F 2018(Probiotics,12 weeks) | 60 | 30 | 30 | 6.20 | 67.15 | 1.20 | 63.18 |
| Tajabadi-Ebrahimi 2017(Probiotics +inulin,12 weeks) | 60 | 30 | 30 | 22.80 | 68.45 | 11.60 | 65.51 |

**CODE：metan tc tfmean tfsd nc cfmean cfsd, label(namevar=author) fixed nostandard**

**CODE：metafunnel _ES _seES**


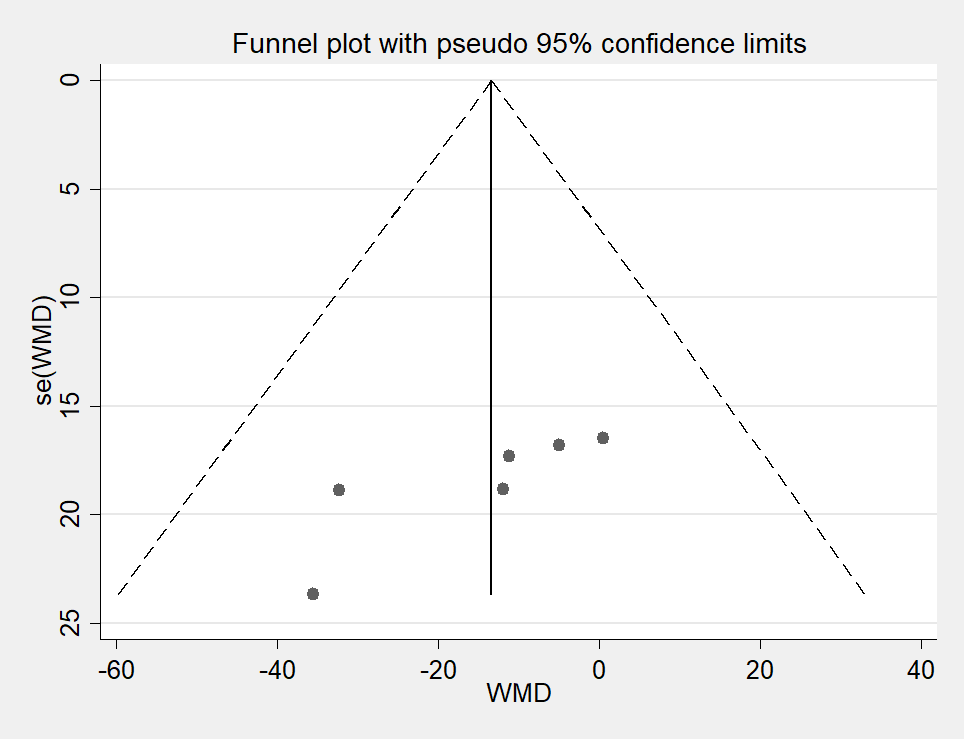


**CODE：metabias6 _ES _seES, graph(egger)**

**CODE：metaninf tc tfmean tfsd nc cfmean cfsd, label(namevar=author) fixed nostandard**


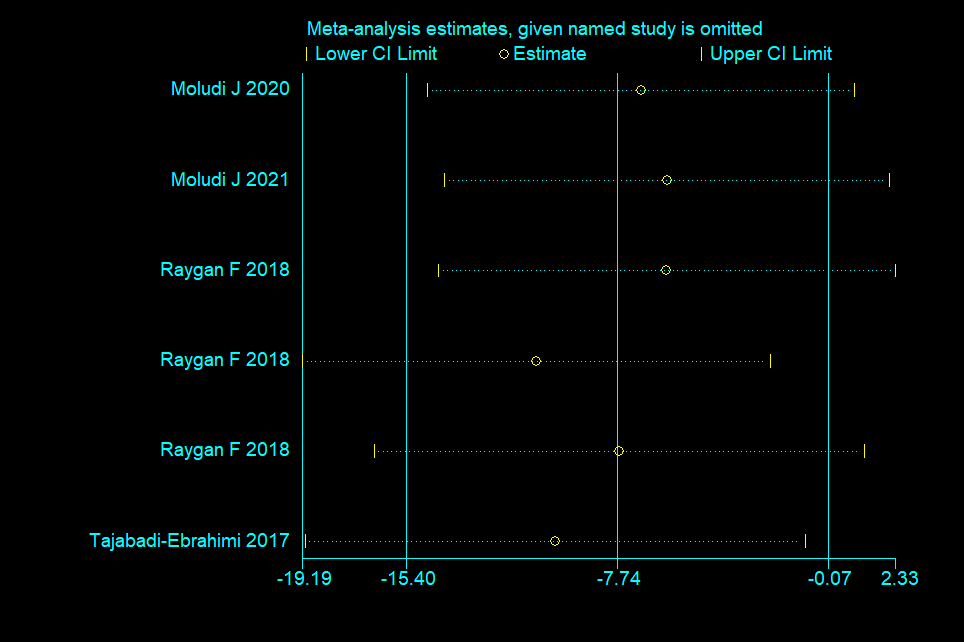


| TC | | | | | | | |
| --- | --- | --- | --- | --- | --- | --- | --- |
| Author | all | nc | tc | cfmean | cfsd | tfmean | tfsd |
| Moludi J 2020(LGG+inlin,2 months) | 48 | 24 | 24 | 16.22 | 92.46 | -26.14 | 81.76 |
| Moludi J 2021(LGG,12weeks) | 44 | 22 | 22 | -6.50 | 44.22 | -30.40 | 37.50 |
| Raygan F 2018(Probiotics+selenlum,12 weeks) | 54 | 27 | 27 | 3.30 | 45.86 | -15.00 | 27.47 |
| Raygan F 2018(Probiotics+vitamin D3,12 weeks) | 60 | 30 | 30 | 0.20 | 31.77 | 5.40 | 38.20 |
| Raygan F 2018(Probiotics,12 weeks) | 60 | 30 | 30 | 2.80 | 32.39 | -5.10 | 26.91 |
| Tajabadi-Ebrahimi 2017(Probiotics +inulin,12 weeks) | 60 | 30 | 30 | 6.70 | 30.90 | 4.60 | 25.41 |

**CODE：metan tc tfmean tfsd nc cfmean cfsd, label(namevar=author) fixed nostandard**

**CODE：metafunnel _ES _seES**


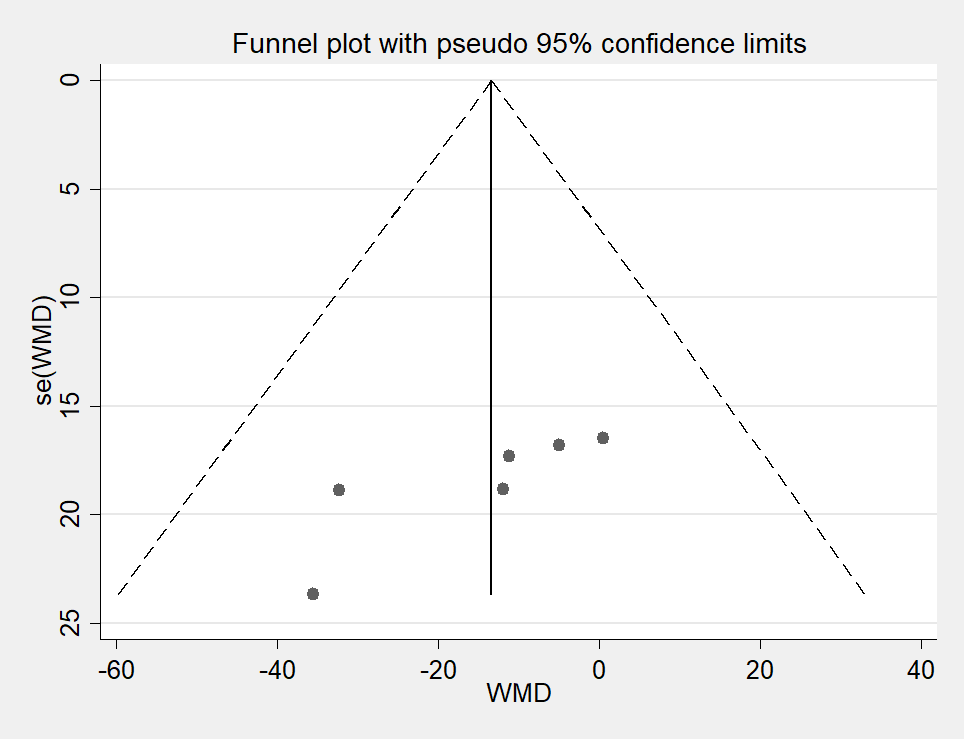


**CODE：metabias6 _ES _seES, graph(egger)**

**CODE：metaninf tc tfmean tfsd nc cfmean cfsd, label(namevar=author) fixed nostandard**


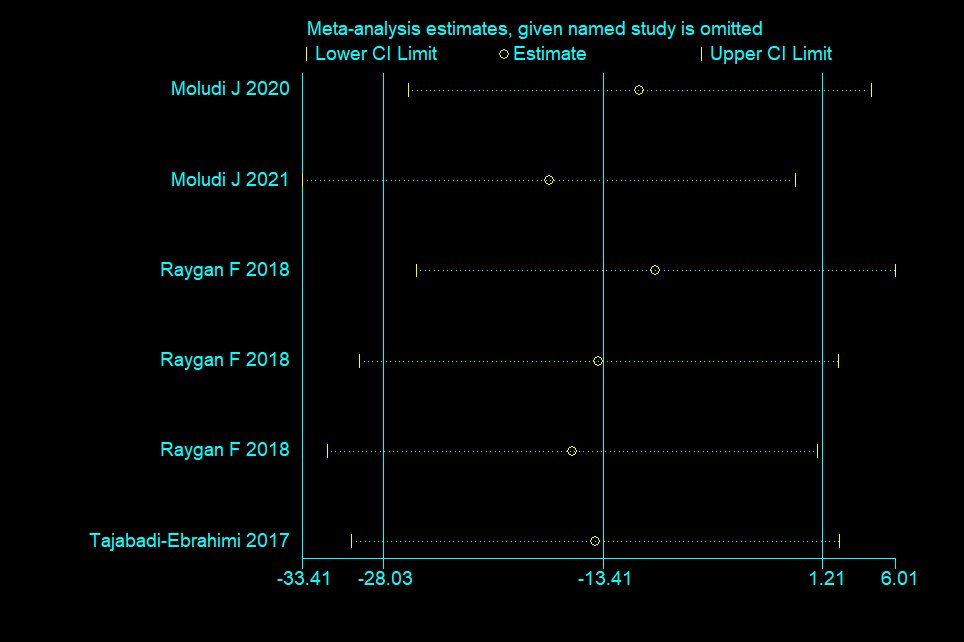


| VLDL | | | | | | | |
| --- | --- | --- | --- | --- | --- | --- | --- |
| Author | all | nc | tc | cfmean | cfsd | tfmean | tfsd |
| Moludi J 2020(LGG+inlin,2 months) | 48 | 24 | 24 | -0.20 | 13.60 | -6.60 | 14.14 |
| Moludi J 2021(LGG,12weeks) | 44 | 22 | 22 | 0.00 | 12.72 | -2.30 | 16.31 |
| Raygan F 2018(Probiotics+selenlum,12 weeks) | 54 | 27 | 27 | 1.30 | 13.45 | 0.30 | 12.66 |
| Raygan F 2018(Probiotics+vitamin D3,12 weeks) | 60 | 30 | 30 | 4.50 | 13.71 | 2.30 | 13.05 |

**CODE：metan tc tfmean tfsd nc cfmean cfsd, label(namevar=author) fixed nostandard**

**CODE：metafunnel _ES _seES**


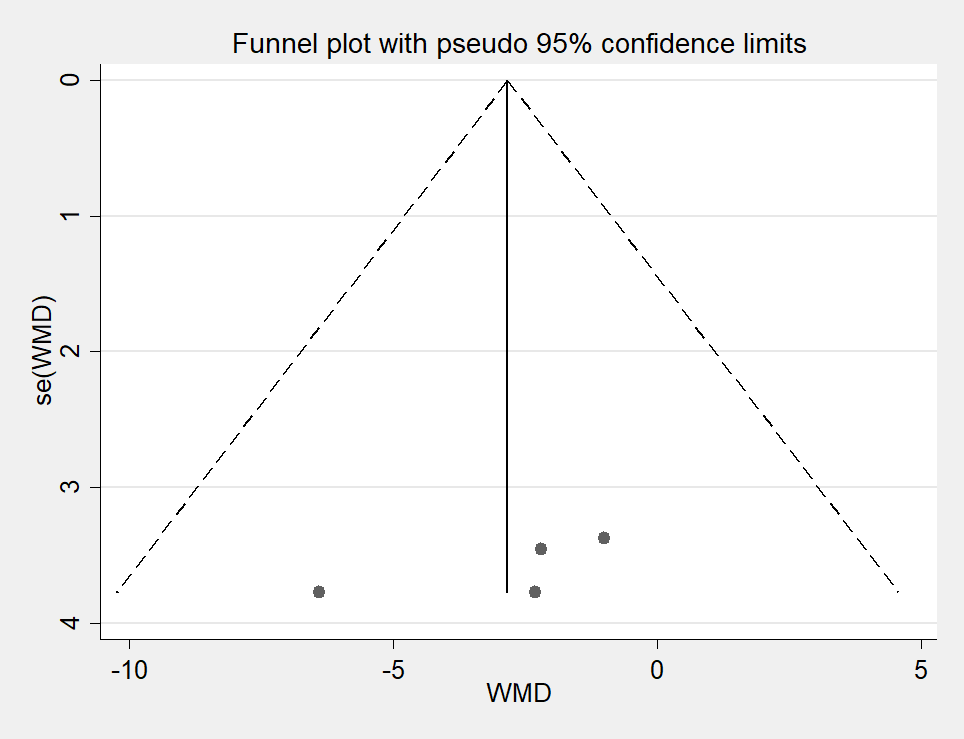


**CODE：metabias6 _ES _seES, graph(egger)**

**CODE：metaninf tc tfmean tfsd nc cfmean cfsd, label(namevar=author) fixed nostandard**


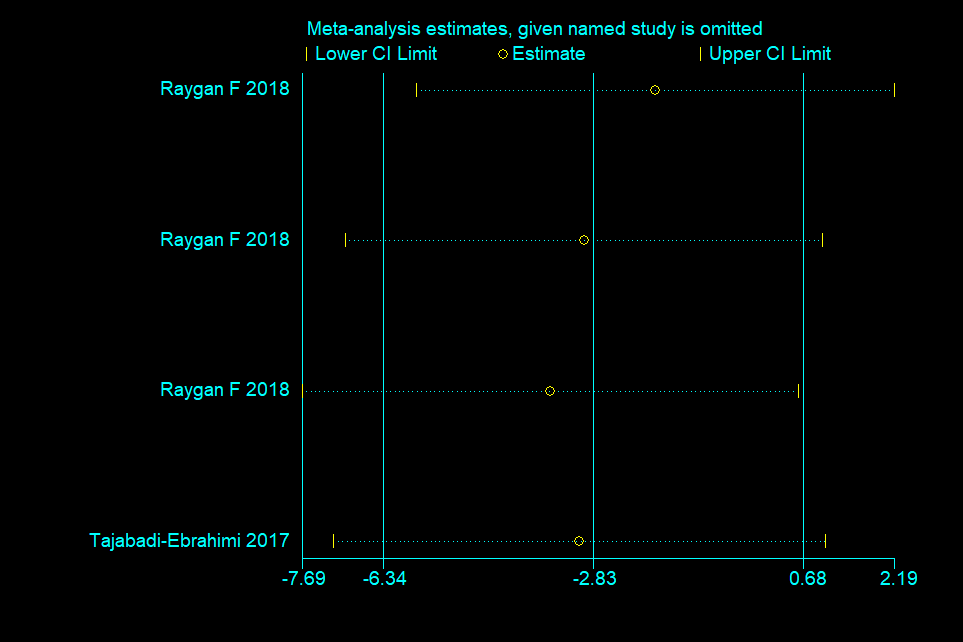


| Total-/HDL-cholesterol ratio | | | | | | | |
| --- | --- | --- | --- | --- | --- | --- | --- |
| Author | all | nc | tc | cfmean | cfsd | tfmean | tfsd |
| Raygan F 2018(Probiotics,12 weeks) | 60 | 30 | 30 | 0.00 | 0.75 | -0.20 | 0.60 |
| Tajabadi-Ebrahimi 2017(Probiotics +inulin,12 weeks) | 60 | 30 | 30 | 0.30 | 0.66 | 0.00 | 0.56 |

**CODE：metan tc tfmean tfsd nc cfmean cfsd, label(namevar=author) fixed nostandard**

**CODE：metafunnel _ES _seES**


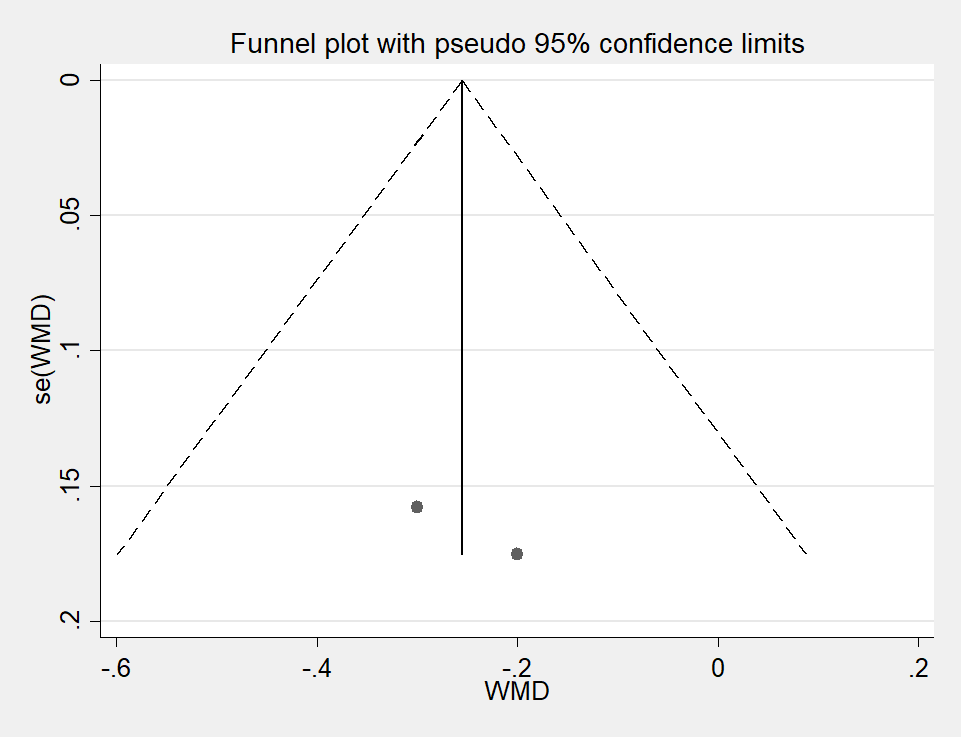


| FPG |  |  |  |  |  |  |  |
| --- | --- | --- | --- | --- | --- | --- | --- |
| Author | all | nc | tc | cfmean | cfsd | tfmean | tfsd |
| Moludi J 2020(LGG+inlin,2 months) | 48 | 24 | 24 | -6.68 | 71.15 | -8.81 | 29.34 |
| Moludi J 2021(LGG,12weeks) | 44 | 22 | 22 | -9.96 | 27.18 | -17.91 | 53.58 |
| Raygan F 2018(Probiotics+selenlum,12 weeks) | 54 | 27 | 27 | -2.1 | 43.51 | -12.50 | 43.81 |
| Raygan F 2018(Probiotics+vitamin D3,12 weeks) | 60 | 30 | 30 | -2.5 | 34.85 | -6.40 | 48.22 |
| Raygan F 2018(Probiotics,12 weeks) | 60 | 30 | 30 | 9.4 | 42.06 | -13.20 | 41.37 |
| Tajabadi-Ebrahimi 2017(Probiotics +inulin,12 weeks) | 60 | 30 | 30 | 19.2 | 64.37 | -19.70 | 53.61 |

**CODE：metan tc tfmean tfsd nc cfmean cfsd, label(namevar=author) fixed nostandard**

**CODE：metafunnel _ES _seES**


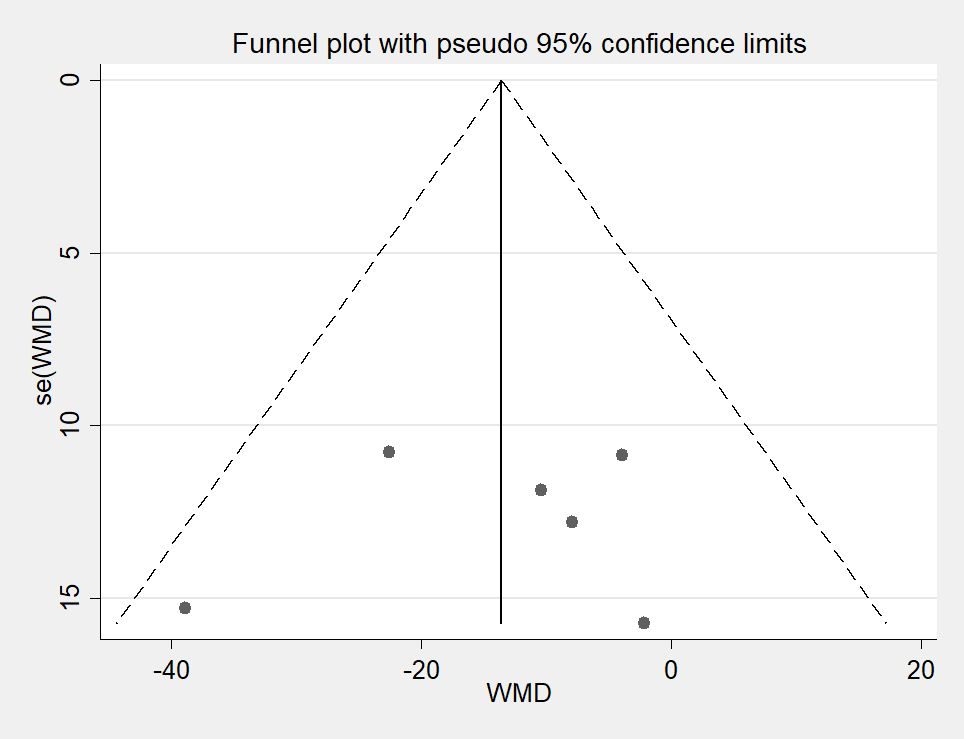


**CODE：metabias6 _ES _seES, graph(egger)**

**CODE：metaninf tc tfmean tfsd nc cfmean cfsd, label(namevar=author) fixed nostandard**


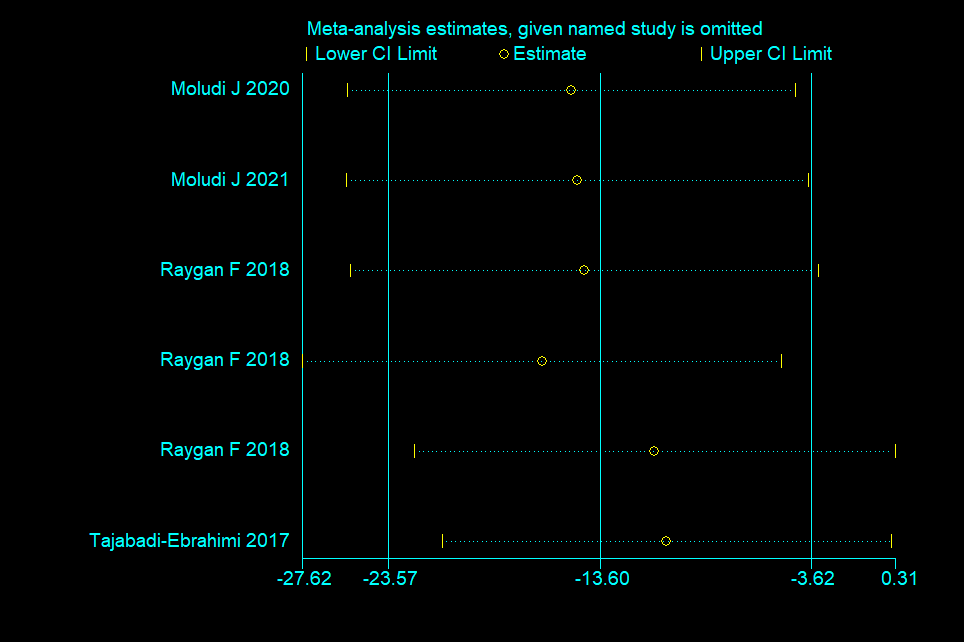


| HOMA-IR |  |  |  |  |  |  |  |
| --- | --- | --- | --- | --- | --- | --- | --- |
| Author | all | nc | tc | cfmean | cfsd | tfmean | tfsd |
| Raygan F 2018(Probiotics+selenlum,12 weeks) | 54 | 27 | 27 | 0.3 | 2.01 | -0.90 | 1.40 |
| Raygan F 2018(Probiotics+vitamin D3,12 weeks) | 60 | 30 | 30 | -0.1 | 2.50 | -1.00 | 3.29 |
| Raygan F 2018(Probiotics,12 weeks) | 60 | 30 | 30 | 0.1 | 3.42 | -0.40 | 2.56 |
| Tajabadi-Ebrahimi 2017(Probiotics +inulin,12 weeks) | 60 | 30 | 30 | 0.9 | 4.10 | 0.00 | 3.11 |

**CODE：metan tc tfmean tfsd nc cfmean cfsd, label(namevar=author) fixed nostandard**

**CODE：metafunnel _ES _seES**


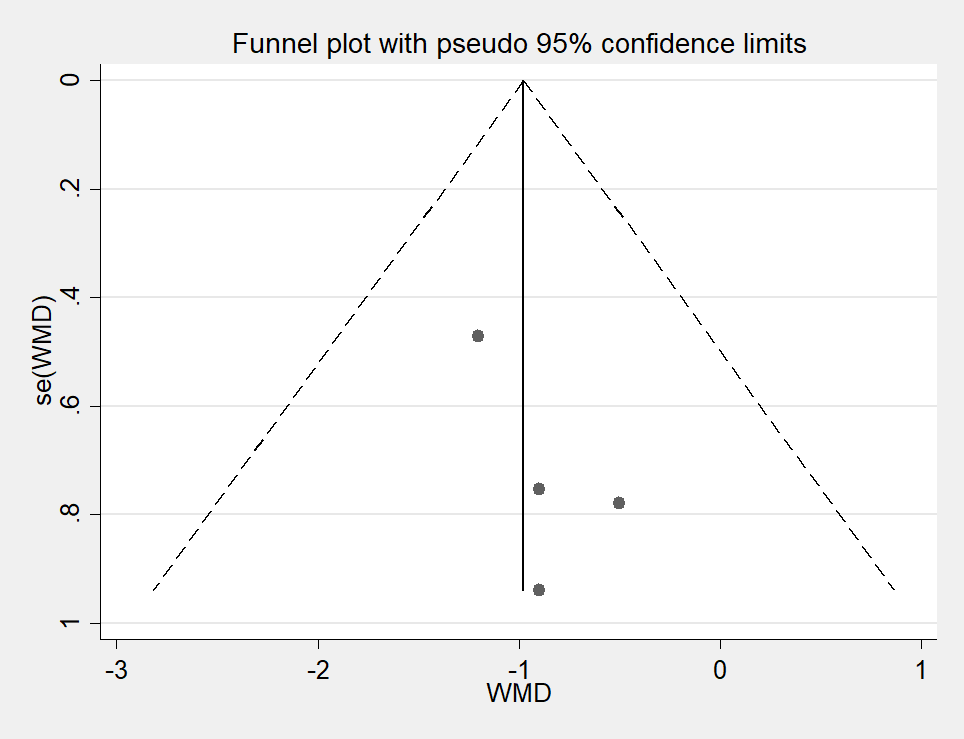


**CODE：metabias6 _ES _seES, graph(egger)**

**CODE：metaninf tc tfmean tfsd nc cfmean cfsd, label(namevar=author) fixed nostandard**


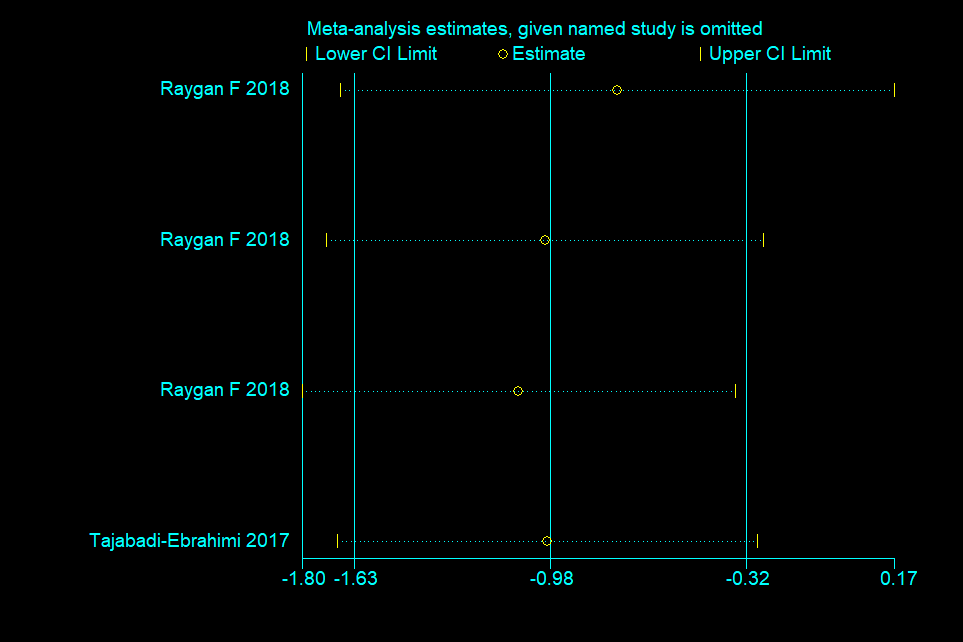


| Insulin |  |  |  |  |  |  |  |
| --- | --- | --- | --- | --- | --- | --- | --- |
| Author | all | nc | tc | cfmean | cfsd | tfmean | tfsd |
| Raygan F 2018(Probiotics+selenlum,12 weeks) | 54 | 27 | 27 | 1.20 | 4.85 | -2.60 | 2.61 |
| Raygan F 2018(Probiotics+vitamin D3,12 weeks) | 60 | 30 | 30 | 0.20 | 7.85 | -2.80 | 7.35 |
| Raygan F 2018(Probiotics,12 weeks) | 60 | 30 | 30 | 0.90 | 8.55 | -1.20 | 5.41 |
| Tajabadi-Ebrahimi 2017(Probiotics +inulin,12 weeks) | 60 | 30 | 30 | 3.30 | 9.62 | -0.70 | 7.95 |

**CODE：metan tc tfmean tfsd nc cfmean cfsd, label(namevar=author) fixed nostandard**

**CODE：metafunnel _ES _seES**


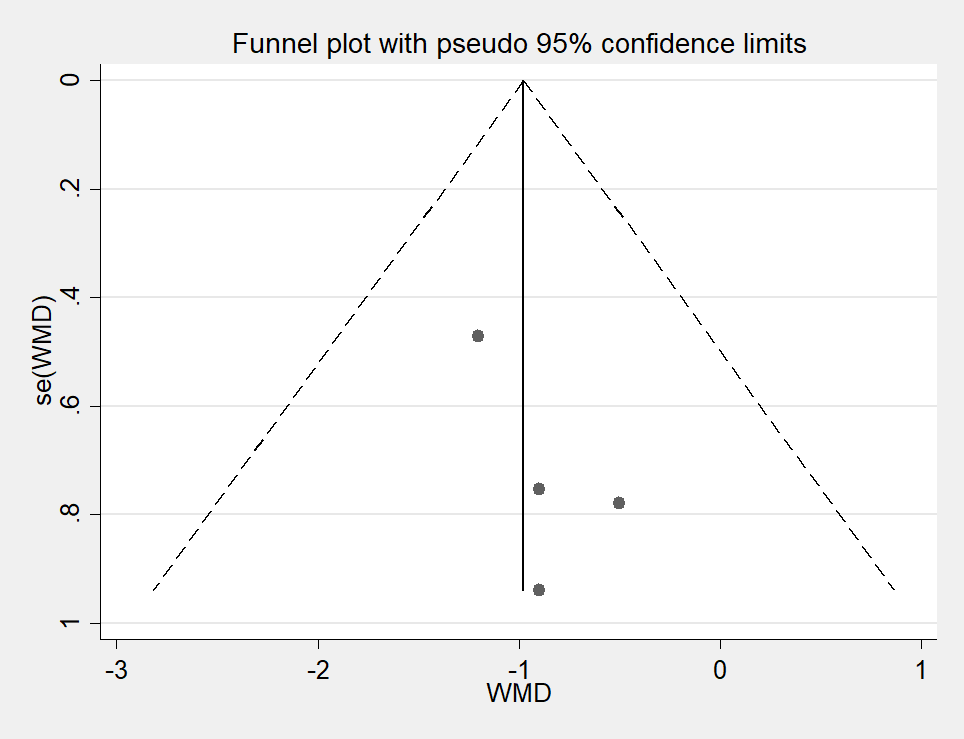


**CODE：metabias6 _ES _seES, graph(egger)**

**CODE：metaninf tc tfmean tfsd nc cfmean cfsd, label(namevar=author) fixed nostandard**


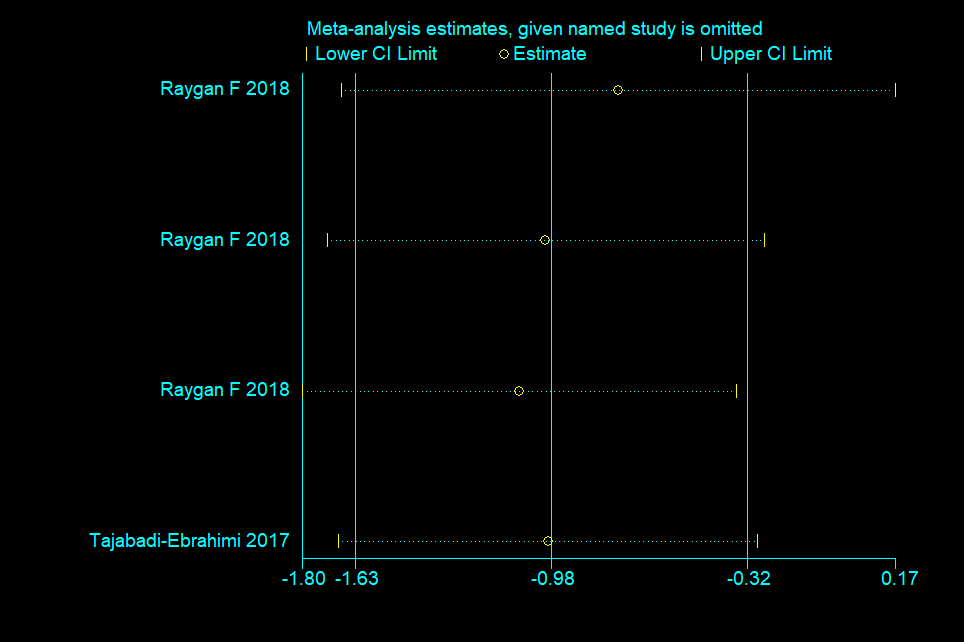


| QUICKI |  |  |  |  |  |  |  |
| --- | --- | --- | --- | --- | --- | --- | --- |
| Author | all | nc | tc | cfmean | cfsd | tfmean | tfsd |
| Raygan F 2018(Probiotics+selenlum,12 weeks) | 54 | 27 | 27 | -0.01 | 0.02 | 0.02 | 0.01 |
| Raygan F 2018(Probiotics+vitamin D3,12 weeks) | 60 | 30 | 30 | 0.00 | 0.02 | 0.03 | 0.05 |
| Raygan F 2018(Probiotics,12 weeks) | 60 | 30 | 30 | -0.01 | 0.03 | 0.00 | 0.02 |
| Tajabadi-Ebrahimi 2017(Probiotics +inulin,12 weeks) | 60 | 30 | 30 | -0.01 | 0.03 | 0.00 | 0.02 |

**CODE：metan tc tfmean tfsd nc cfmean cfsd, label(namevar=author) random nostandard**

**CODE：metafunnel _ES _seES**


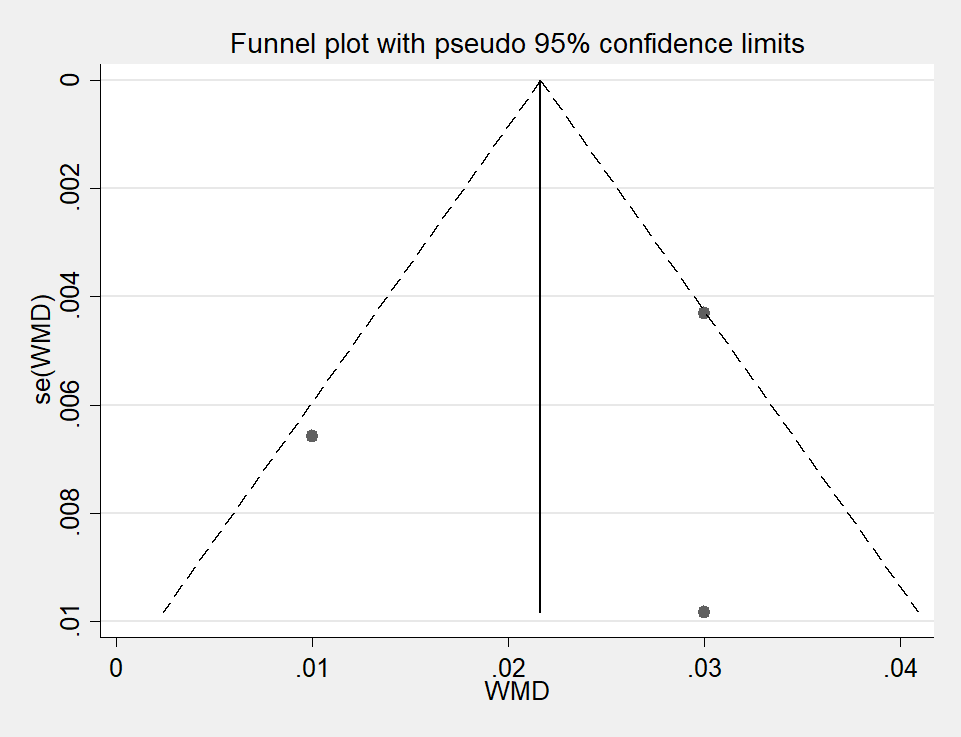


**CODE：metabias6 _ES _seES, graph(egger)**

**CODE：metaninf tc tfmean tfsd nc cfmean cfsd, label(namevar=author) fixed nostandard**


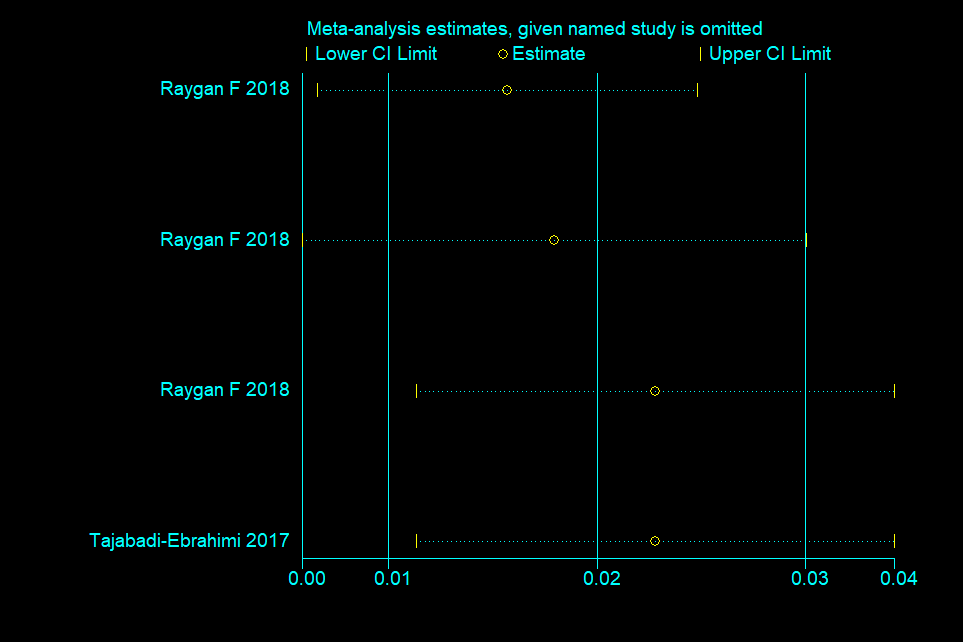


| DBP |  |  |  |  |  |  |  |
| --- | --- | --- | --- | --- | --- | --- | --- |
| Author | all | nc | tc | cfmean | cfsd | tfmean | tfsd |
| Moludi J 2020(LGG+inlin,2 months) | 48 | 24 | 24 | 1.64 | 21.80 | -0.62 | 13.71 |
| Moludi J 2021(LGG,12weeks) | 44 | 22 | 22 | -0.56 | 13.80 | -4.42 | 9.68 |
| Raygan F 2018(Probiotics+selenlum,12 weeks) | 54 | 27 | 27 | 0.30 | 5.42 | 0.70 | 6.48 |
| Raygan F 2018(Probiotics+vitamin D3,12 weeks) | 60 | 30 | 30 | -1.30 | 8.06 | -0.70 | 7.10 |
| Raygan F 2018(Probiotics,12 weeks) | 60 | 30 | 30 | -1.10 | 8.65 | -1.70 | 7.51 |

**CODE：metan tc tfmean tfsd nc cfmean cfsd, label(namevar=author) fixed nostandard**

**CODE：metafunnel _ES _seES**


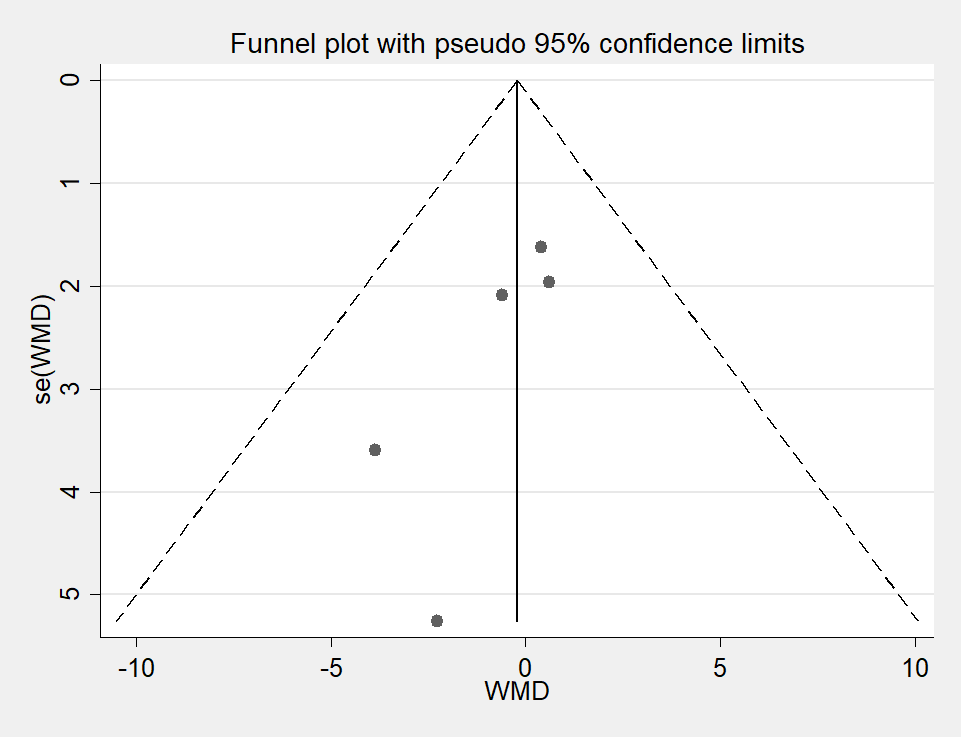


**CODE：metabias6 _ES _seES, graph(egger)**

**CODE：metaninf tc tfmean tfsd nc cfmean cfsd, label(namevar=author) fixed nostandard**


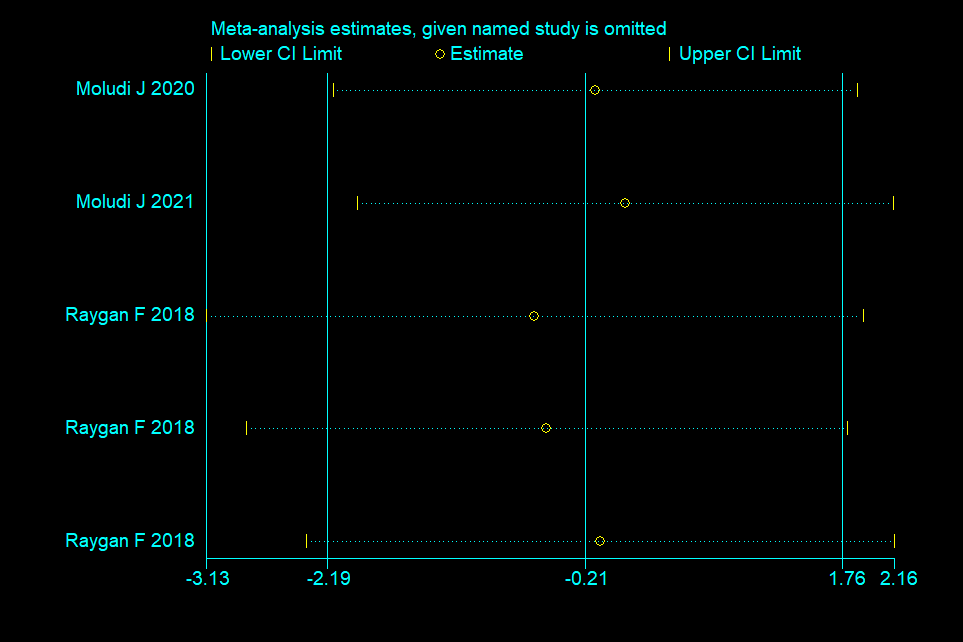


| SBP |  |  |  |  |  |  |  |
| --- | --- | --- | --- | --- | --- | --- | --- |
| Author | all | nc | tc | cfmean | cfsd | tfmean | tfsd |
| Moludi J 2020(LGG+inlin,2 months) | 48 | 24 | 24 | -4.46 | 17.15 | -7.42 | 22.72 |
| Moludi J 2021(LGG,12weeks) | 44 | 22 | 22 | -2.44 | 15.45 | -7.70 | 17.44 |
| Raygan F 2018(Probiotics+selenlum,12 weeks) | 54 | 27 | 27 | -0.80 | 8.58 | -1.80 | 11.40 |
| Raygan F 2018(Probiotics+vitamin D3,12 weeks) | 60 | 30 | 30 | -1.00 | 9.28 | -0.70 | 8.16 |
| Raygan F 2018(Probiotics,12 weeks) | 60 | 30 | 30 | -1.30 | 14.76 | -1.90 | 13.06 |

**CODE：metan tc tfmean tfsd nc cfmean cfsd, label(namevar=author) fixed nostandard**

**CODE：metafunnel _ES _seES**


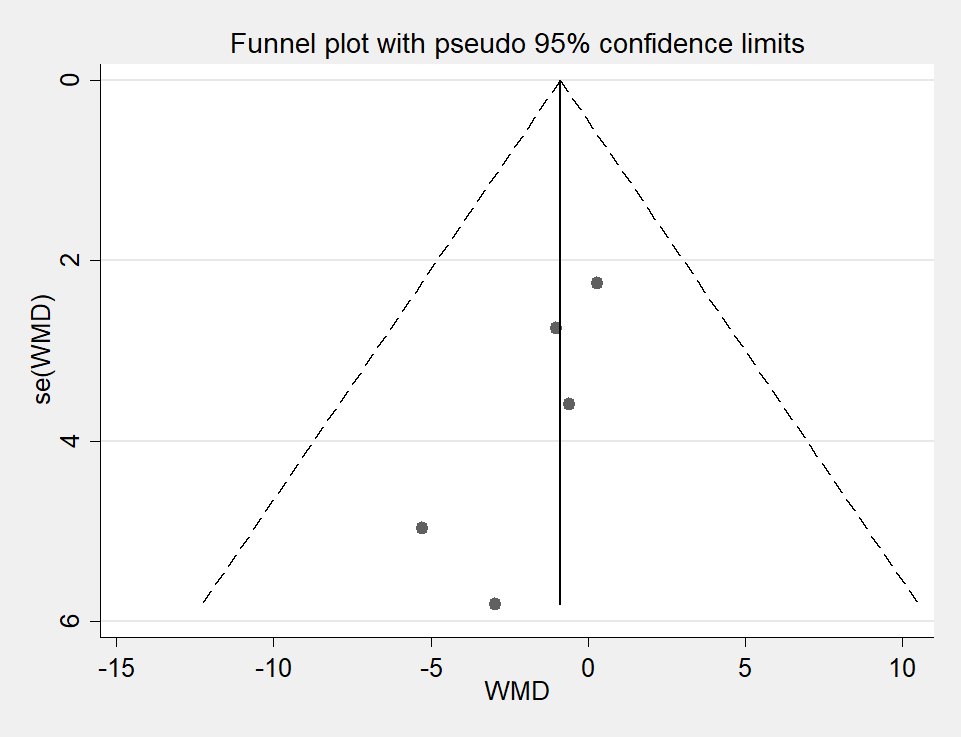


**CODE：metabias6 _ES _seES, graph(egger)**

**CODE：metaninf tc tfmean tfsd nc cfmean cfsd, label(namevar=author) fixed nostandard**


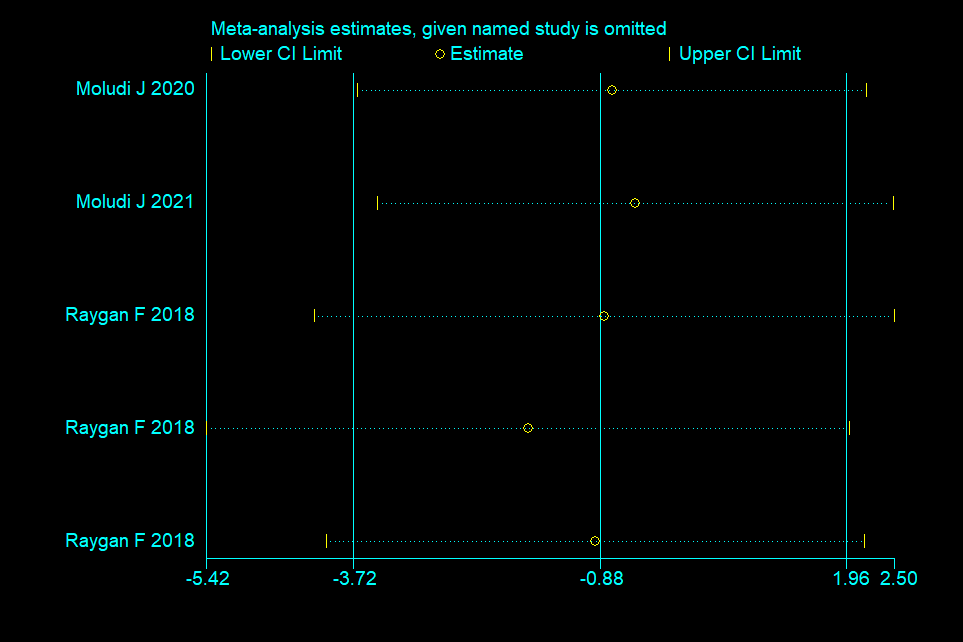


| NO |  |  |  |  |  |  |  |
| --- | --- | --- | --- | --- | --- | --- | --- |
| Author | all | nc | tc | cfmean | cfsd | tfmean | tfsd |
| Raygan F 2018(Probiotics+selenlum,12 weeks) | 54 | 27 | 27 | -0.60 | 8.51 | 7.00 | 5.91 |
| Raygan F 2018(Probiotics+vitamin D3,12 weeks) | 60 | 30 | 30 | -1.40 | 8.46 | 1.70 | 3.64 |
| Raygan F 2018(Probiotics,12 weeks) | 60 | 30 | 30 | -2.40 | 9.27 | 4.10 | 6.88 |

**CODE：metan tc tfmean tfsd nc cfmean cfsd, label(namevar=author) fixed nostandard**

**CODE：metafunnel _ES _seES**


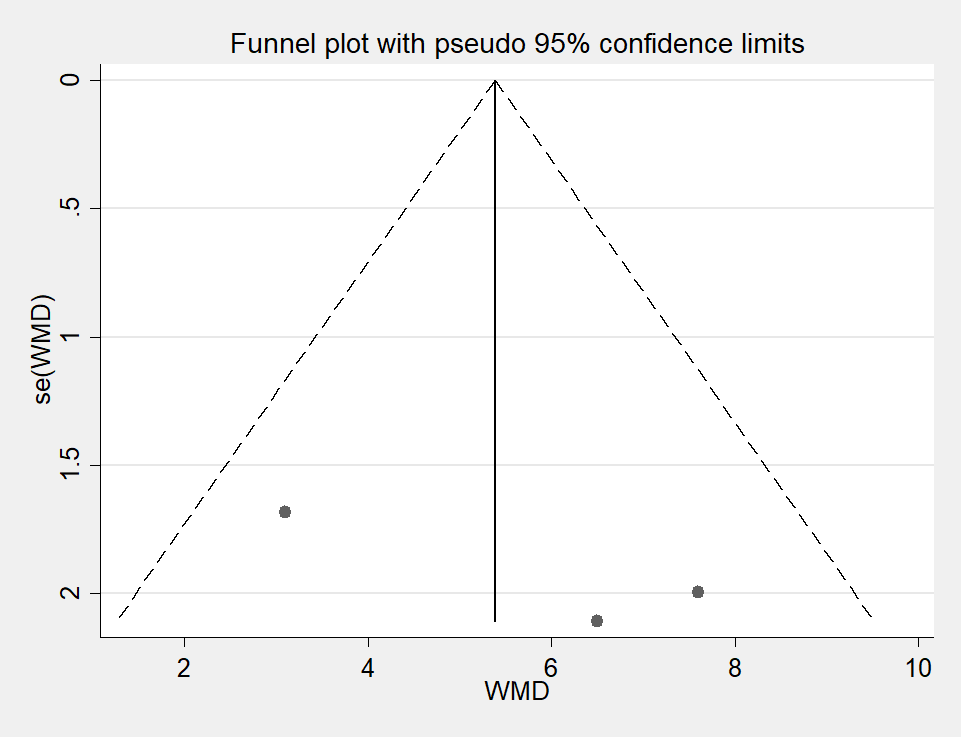


**CODE：metabias6 _ES _seES, graph(egger)**

**CODE：metaninf tc tfmean tfsd nc cfmean cfsd, label(namevar=author) fixed nostandard**


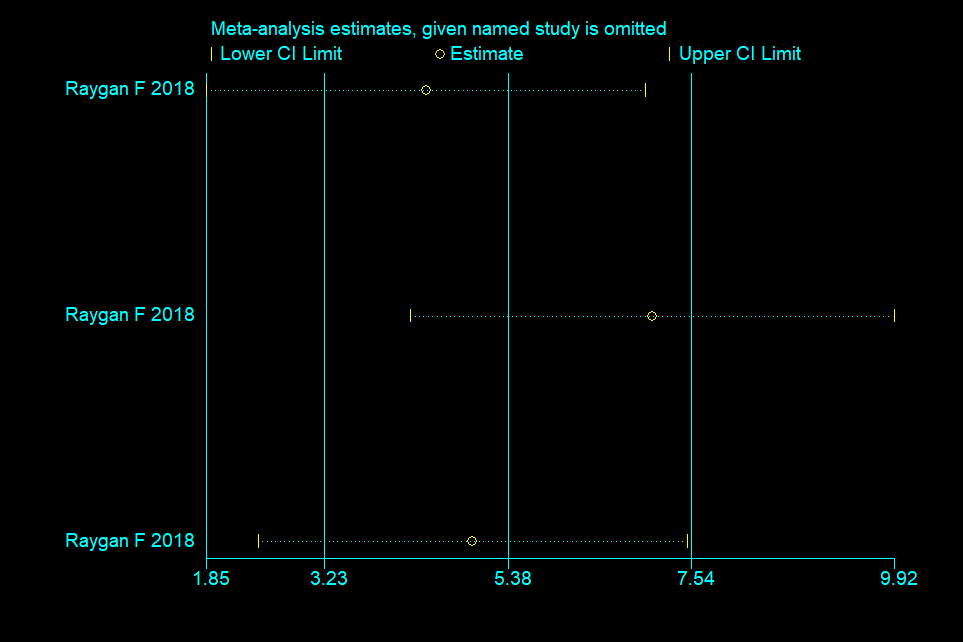


| GSH |  |  |  |  |  |  |  |
| --- | --- | --- | --- | --- | --- | --- | --- |
| Author | all | nc | tc | cfmean | cfsd | tfmean | tfsd |
| Raygan F 2018(Probiotics+selenlum,12 weeks) | 54 | 27 | 27 | -19.80 | 149.20 | 127.20 | 129.82 |
| Raygan F 2018(Probiotics+vitamin D3,12 weeks) | 60 | 30 | 30 | -12.20 | 144.07 | 18.00 | 123.92 |
| Raygan F 2018(Probiotics,12 weeks) | 60 | 30 | 30 | -1.00 | 102.68 | 43.20 | 163.30 |

**CODE：metan tc tfmean tfsd nc cfmean cfsd, label(namevar=author) random cohen**

**CODE：metafunnel _ES _seES**


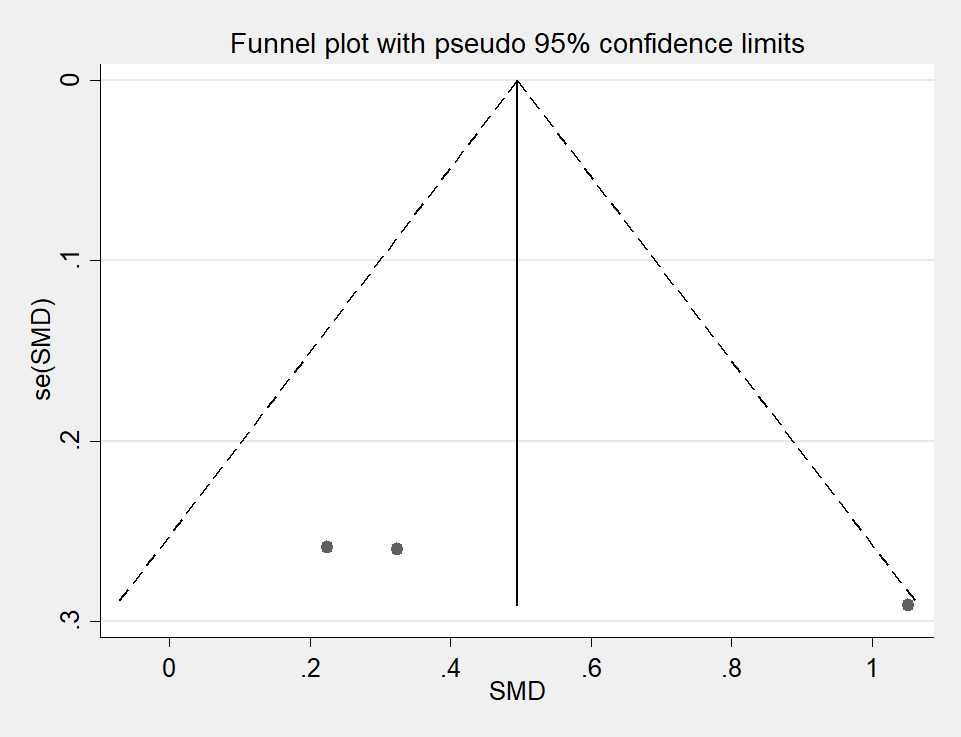


**CODE：metabias6 _ES _seES, graph(egger)**

**CODE：metaninf tc tfmean tfsd nc cfmean cfsd, label(namevar=author) random cohen**


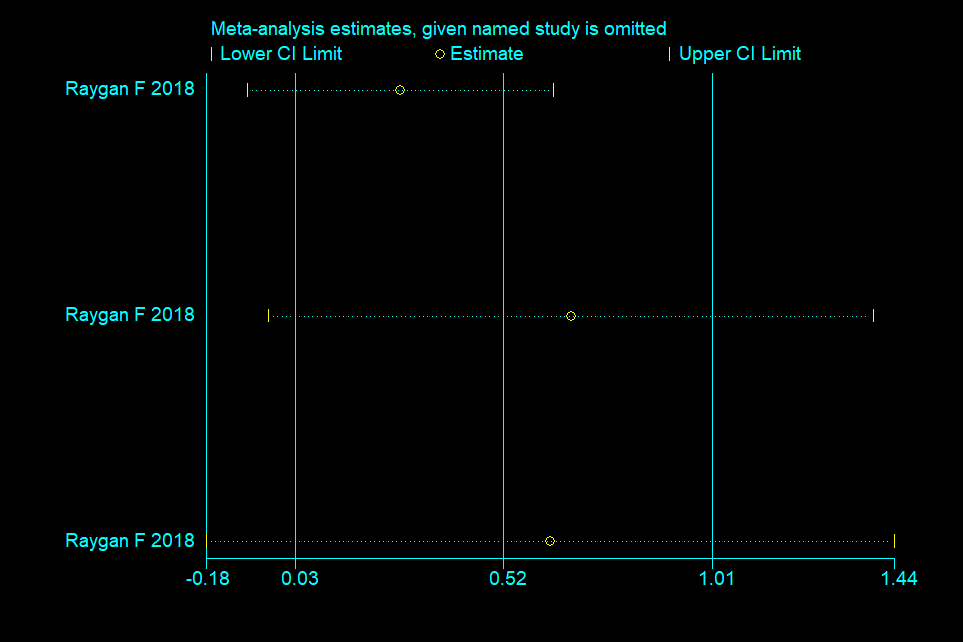


| TAC |  |  |  |  |  |  |  |
| --- | --- | --- | --- | --- | --- | --- | --- |
| Author | all | nc | tc | cfmean | cfsd | tfmean | tfsd |
| Raygan F 2018(Probiotics+selenlum,12 weeks) | 54 | 27 | 27 | -15.10 | 199.57 | 79.60 | 100.68 |
| Raygan F 2018(Probiotics+vitamin D3,12 weeks) | 60 | 30 | 30 | -117.00 | 333.97 | 12.60 | 91.55 |
| Raygan F 2018(Probiotics,12 weeks) | 60 | 30 | 30 | -22.00 | 289.95 | 78.90 | 247.51 |

**CODE：metan tc tfmean tfsd nc cfmean cfsd, label(namevar=author) fixed nostandard**

**CODE：metafunnel _ES _seES**


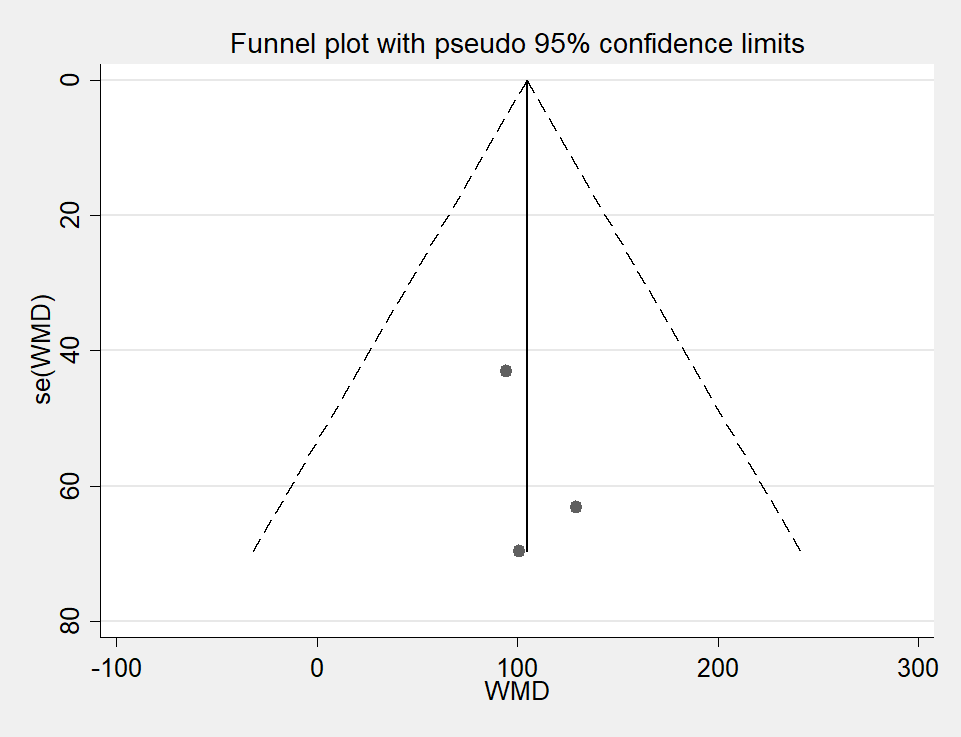


**CODE：metabias6 _ES _seES, graph(egger)**

**CODE：metaninf tc tfmean tfsd nc cfmean cfsd, label(namevar=author) fixed nostandard**


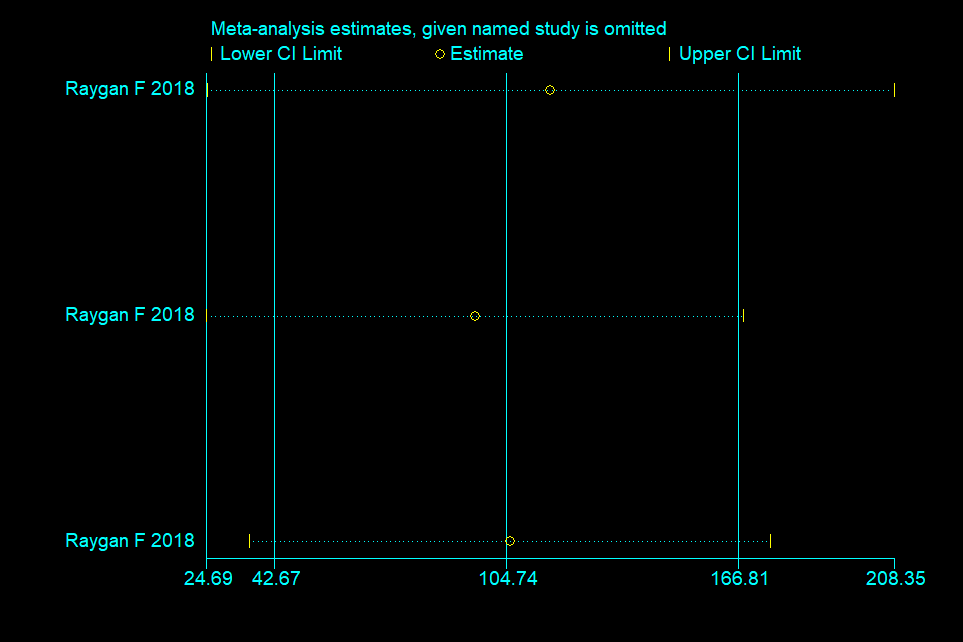


| hs-CRP |  |  |  |  |  |  |  |
| --- | --- | --- | --- | --- | --- | --- | --- |
| Author | all | nc | tc | cfmean | cfsd | tfmean | tfsd |
| Moludi J 2020(LGG+inlin,2 months) | 48 | 24 | 24 | 0.82 | 2.90 | -1.69 | 1.58 |
| Moludi J 2021(LGG,3 months) | 44 | 22 | 22 | -0.65 | 1.11 | -1.45 | 1.30 |
| Moludi J 2021(LGG,12weeks) | 44 | 22 | 22 | -0.67 | 1.53 | -1.75 | 1.53 |
| Raygan F 2018(Probiotics+selenlum,12 weeks) | 54 | 27 | 27 | 637.70 | 2359.13 | -485.20 | 1282.97 |
| Raygan F 2018(Probiotics+vitamin D3,12 weeks) | 60 | 30 | 30 | 260.50 | 2982.92 | -950.00 | 2270.62 |
| Raygan F 2018(Probiotics,12 weeks) | 60 | 30 | 30 | 0.10 | 2.55 | -0.80 | 2.71 |

**CODE：metan tc tfmean tfsd nc cfmean cfsd, label(namevar=author) fixed cohen**

**CODE：metafunnel _ES _seES**


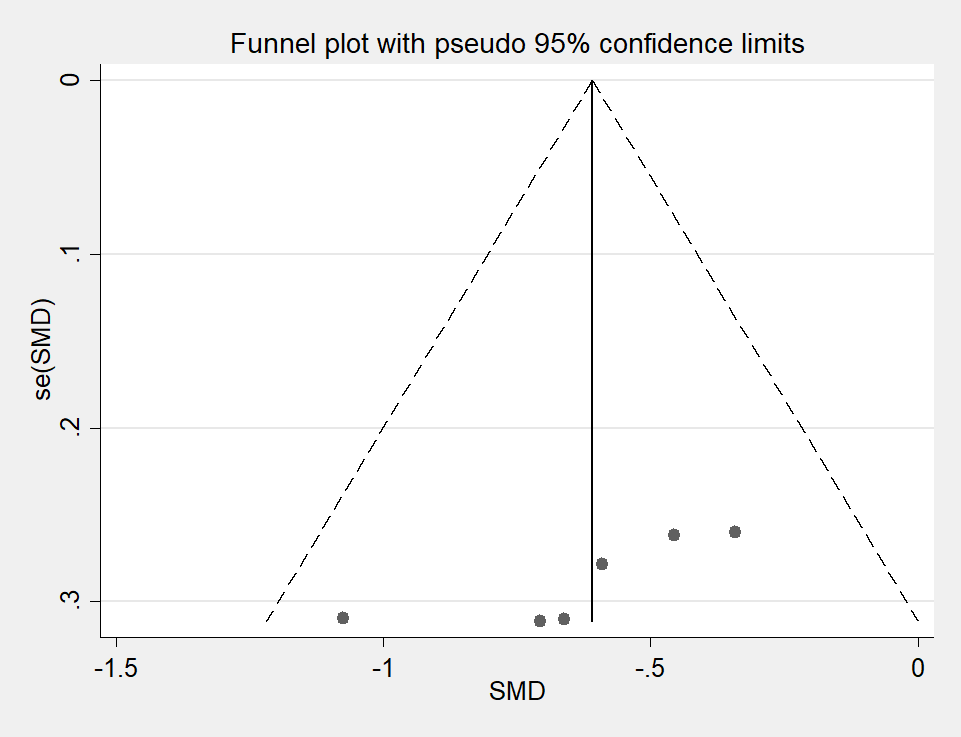


**CODE：metabias6 _ES _seES, graph(egger)**

**CODE：metaninf tc tfmean tfsd nc cfmean cfsd, label(namevar=author) fixed cohen**


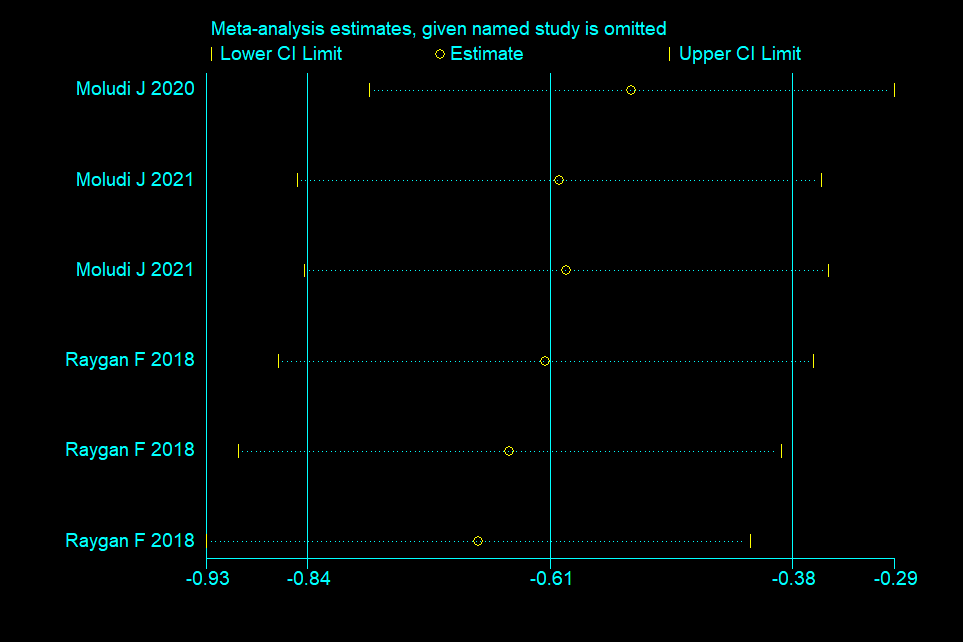


| TMAO |  |  |  |  |  |  |  |
| --- | --- | --- | --- | --- | --- | --- | --- |
| Author | all | nc | tc | cfmean | cfsd | tfmean | tfsd |
| Sun, B 2022(Probio M8 ,6 months ) | 60 | 24 | 36 | 15.41 | 51.58 | -9.97 | 24.77 |
| Moludi J 2021(LGG,3 months) | 44 | 22 | 22 | -5.09 | 31.89 | -17.26 | 16.27 |

**CODE：metan tc tfmean tfsd nc cfmean cfsd, label(namevar=author) fixed cohen**

**CODE：metafunnel _ES _seES**


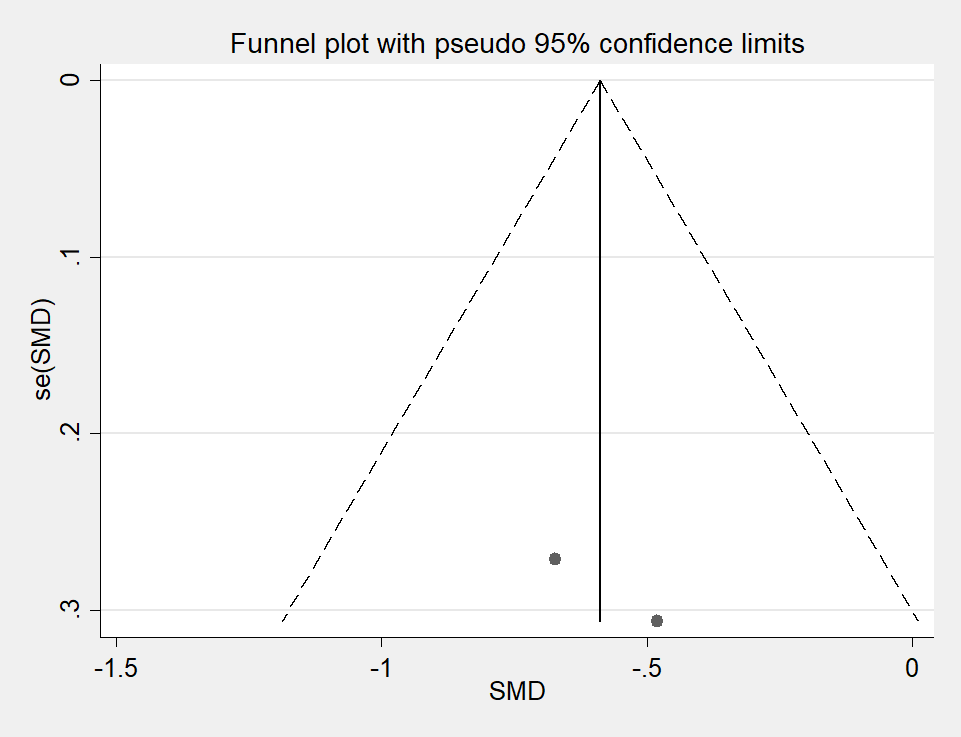


| LPS |  |  |  |  |  |  |  |
| --- | --- | --- | --- | --- | --- | --- | --- |
| Author | all | nc | tc | cfmean | cfsd | tfmean | tfsd |
| Moludi J 2020(LGG+inlin,2 months) | 48 | 24 | 24 | 0.31 | 26.52 | -22.02 | 23.49 |
| Moludi J 2021(LGG,12weeks)2 | 44 | 22 | 22 | -2.96 | 15.08 | -5.88 | 10.13 |

**CODE：metan tc tfmean tfsd nc cfmean cfsd, label(namevar=author) random cohen**

**CODE：metafunnel _ES _seES**


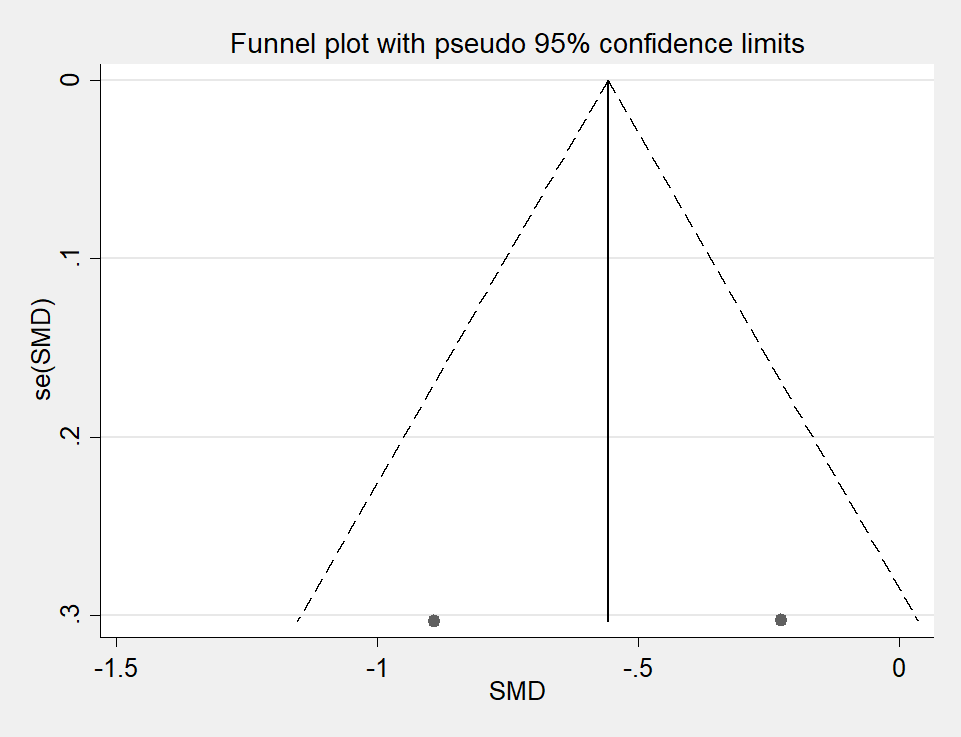

Supplement: Supplementary file 7 [file Datasheet1.docx]
